# The RBPJ/DAPK3/UBE3A signaling axis induces PBRM1 degradation to modulate the sensitivity of renal cell carcinoma to CDK4/6 inhibitors

**DOI:** 10.1038/s41419-022-04760-6

**Published:** 2022-04-02

**Authors:** Wentao Liu, Bin Zhang, Dan Zhang, Feng Guo, Kun Ye, Liang Zhu, Xin Jin

**Affiliations:** 1 Department of Urology, The Second Xiangya Hospital, Central South University, 410011 Changsha, Hunan China; 2 Cancer center, Union Hospital, Tongji Medical College, Huazhong University of Science and Technology, 430022 Wuhan, China; 3 Uro-Oncology Institute of Central South University, 430022 Changsha, Hunan China

**Keywords:** Renal cell carcinoma, Oncogenes

## Abstract

Renal cell carcinoma (RCC) is a kind of malignant tumor originating from the renal tubular epithelium. Approximately 30% of patients with renal cancer are found to have metastasis when first diagnosed. Exploring other effective treatment methods in addition to surgery is an urgent need in the research field of renal cell carcinoma. Polybromo 1 (PBRM1) is the second most mutated gene in RCC, with a mutation rate of ~40%. Notably, the posttranscriptional modification of PBRM1 in RCC is unclear. In this study, we performed unbiased mass spectrometry of PBRM1 and identified ubiquitin-protein ligase E3A (UBE3A), an extensively studied E3 ligase that can bind with PBRM1 and regulate the stability of PBRM1 in renal cancer cells. We further found that RBPJ/DAPK3 modulated the E3 ligase activity of UBE3A by interfering with the PKA phosphorylation of UBE3A. Finally, we demonstrated that the RBPJ/DAPK3/UBE3A/PBRM1/p21 axis contributed to the sensitivity of renal cancer cells to CDK4/6 inhibitors. In addition, in combination with RBPJ inhibitors, CDK4/6 inhibitors showed synergistically enhanced effects on renal cancer cells. In summary, we not only revealed a novel RBPJ/DAPK3/UBE3A/PBRM1/p21 signaling axis but also identified a combination strategy for overcoming the resistance of renal cancer cells to CDK4/6 inhibitors.

## Introduction

Renal cell carcinoma (RCC) is a kind of malignant tumor originating from renal tubular epithelium [1]. Approximately 338,000 patients are newly diagnosed with renal cancer every year worldwide, and nearly 30% of these patients have metastasis when first diagnosed [2]. In recent years, the wide application of minimally invasive surgery, such as pure laparoscopic or robot-assisted renal tumor resection or thermal ablation, has greatly improved the tumor control rate and survival rate of patients with early renal cell carcinoma [3]. Currently, systemic antiangiogenetic target therapy significantly improves the prognosis of patients with advanced renal clear cell carcinoma [4]. However, antiangiogenetic drugs cannot achieve complete remission in patients with RCC, and long-term use can lead to a decrease in drug sensitivity [5]. Therefore, the development of new targeted drugs will be helpful in prolonging the survival time of patients with advanced renal cell carcinoma.

Studies have found that polybromo 1 (PBRM1) is the second most mutated gene, with an ~40% mutation rate, in RCC after *VHL* (with a mutation rate of ~75%) [6, 7]. PBRM1 gene mutation is closely related to the occurrence and development of renal cell carcinoma [6]. However, the post-translational modification of PBRM1 in RCC is still unknown.

In this study, we performed unbiased mass spectrometry of PBRM1 and identified ubiquitin-protein ligase E3A (UBE3A), a well-known E3 ligase [8], that can bind with PBRM1 and regulate the stability of PBRM1 in renal cancer cells. We further found that RBPJ/DAPK3 modulated the E3 ligase activity of UBE3A by interfering with the PKA phosphorylation of UBE3A. Finally, we demonstrated that the RBPJ/DAPK3/UBE3A/PBRM1/p21 axis contributed to the sensitivity of renal cancer cells to CDK4/6 inhibitors.

## Materials and methods

### Cell lines and cell culture

The 786-O (CL-0010), ACHN (CL-0021) human renal cancer cell lines, and 293T cells (CL-0005) were purchased from Procell Life Science & Technology (Wuhan, China). All cells were subjected to STR authentication by Procell Life Science & Technology and tested for mycoplasma contamination. The 786-O cells were cultured with RPMI-1640 (PM150110, Procell Life Science & Technology) plus 10% fetal bovine serum (FBS) (164210-500, Procell Life Science & Technology) and 1% penicillin/streptomycin (P/S) (PB180120, Procell Life Science & Technology). The ACHN cells were maintained with MEM (PM150410, Procell Life Science & Technology) supplemented with 10% FBS (164210-500, Procell Life Science & Technology) and 1% P/S (PB180120, Procell Life Science & Technology). The 293T cells were cultured in DMEM (PM150210, Procell Life Science & Technology) supplemented with 10% FBS and 1% P/S. All of these cells were maintained in 5% CO_2_ at 37 °C.

### Plasmids and reagents

Plasmids UBE3A, DAPK3, and PBRM1 were obtained from WZ Bioscience (Shandong, China) and GeneChem (Shanghai, China). Flag-UBE3A was cloned into the CMV-MCS-3xFlag-SV40-neomycin vector by GENECHEM (Shanghai, China). Flag-PBRM1 was cloned into the pEnter vector with C-terminal Flag and His tag by WZ Bioscience (Shandong, China). HA-PBRM1 was cloned into the pCMV-N-HA vector. The cDNA of DAPK3 was obtained from WZ Bioscience (Shandong, China) and cloned into the pCMV-N-Myc vector. pGEX-4T-1 vector was used to generate GST-tag plasmids. The UBE3A mutant was constructed following the instructions of a KOD-plus-mutagenesis kit (SMK-101B, TOYOBO, Japan). The mutagenesis sequencing data for flag-UBE3A-T508A and other mutagenesis were provided in Supplementary fig. 1. Short hairpin RNAs (shRNAs) were obtained from GeneCopoeia (USA), and the sequences of the shRNAs were provided in Table S1. Lipofectamine 2000 purchased from Thermo Fisher Scientific, Shanghai, China) was used to transfect these plasmids and shRNAs. The overexpression of proteins (transfection efficiency) and knockdown of genes was consistent across all three biological replicates.

### Western blotting and co-immunoprecipiation (IP)

For western blotting analysis, collected cell precipitates were added with 100 uL RIPA protein lysate containing 10 uL protease inhibitor on ice for at least 30 min. The BCA protein quantitative kit (P0011, Beyotime, Shanghai, China) was used to measure the protein concentration, and the protein standard curve was read at 570 nm with the enzyme label analyzer to calculate the protein concentration of the sample. The same amount of protein was separated by 10% SDS-PAGE and then transferred to the PVDF membrane. The membrane was sealed with 5% non-fat dry milk in 0.2% Tween-20 in Tris-buffered saline (TBST) for 1 h at room temperature and then probed with primary antibody for 24 h at 4 °C. The secondary antibody combined with horseradish peroxides was incubated, and immunoreactivity was detected. For co-immunoprecipiation (IP), collected cell precipitates were added with 1000 uL RIPA protein lysate containing 100 uL protease inhibitor on ice for at least 30 min. The supernatant was collected and co-cultured with Protein A and G Agarose beads (P2055, Beyotime, Shanghai, China) and primary antibodies or IgG for 24 h at 4 °C. Then, the beads were washed with 1×TBST six times, added with 60 uL sample loading buffer, and boiled for 5 min. Then, these samples were subjected to western blotting analysis. The antibodies used as follows: UBE3A (10344-1-AP, Proteintech; 1:1000 dilution), PBRM1 (12563-1-AP, Proteintech; 1:500 dilution), DAPK3 (2928, Cell signaling technology, 1:1000 dilution), RBPJ (14613-1-AP, Proteintech; 1:1000 dilution); P21 (10355-1-AP, Proteintech; 1:1000 dilution); GAPDH (10494-1-AP, Proteintech; 1:10000 dilution).

### Tumor growth analysis

Ethical approval was obtained by the Ethics Committee of the Second Xiangya Hospital, Xiangya Medical College, Central South University for all animal procedures. Power analysis was used to calculate the sample size required for animal experiments and animals were randomized to different groups. BALB/c-nude mice (4–5 weeks old, 18–20 g) were obtained from Vitalriver (Beijing, China). Renal cancer cells (786-O, 1 × 10^7^ per mouse) with different treatments as indicated in the main text section were subcutaneously injected into the back of mice. The procedure of xenografts assay is described previously [9]. At the study endpoint, the volume and mass of xenografts were measured. The CDK4/6 inhibitors (Palbociclib, HY-50767S, MedChemExpress, China, 200 mg per kg bodyweight, oral administration) and RBPJ Inhibitor-1 (RIN1, HY-137471, MedChemExpress, China, 40 mg per kg bodyweight, intraperitoneal injection) were used for mice study.

### Tissue microarray and immunohistochemistry (IHC)

Tissue microarray (U081ki01, Bioaitech, CN) and IHC were performed to assess the levels of UBE3A (10344-1-AP, Proteintech; 1:500 dilution) and PBRM1 (12563-1-AP, Proteintech; 1:1000 dilution) in renal cancer. The IHC score was evaluated as previously reported [9].

### Statistical analysis

For quantitative reverse transcription PCR (RT-qPCR), Chromatin immunoprecipitation- qPCR (ChIP-qPCR), and MTS assay, experiments were performed in three independent biological replicates, these results were presented with means ± standard error mean (SEM) with the consolidated data from three independent biological replicates. The other data were also expressed as the means ± SEM. Statistical significance was determined using Student’s *t*-test, one-way ANOVA, or two-way ANOVA. Statistical analyses were conducted using GraphPad Prism 5 software. *P*-values <0.05 were considered statistically significant.

Other methods were provided in the Supplementary Information, and sequences of gene-specific shRNAs were provided in Supplementary Table 1.

## Results

### UBE3A binds PBRM1 to decrease the PBRM1 protein level in renal cancer cells

**Fig. 1:**
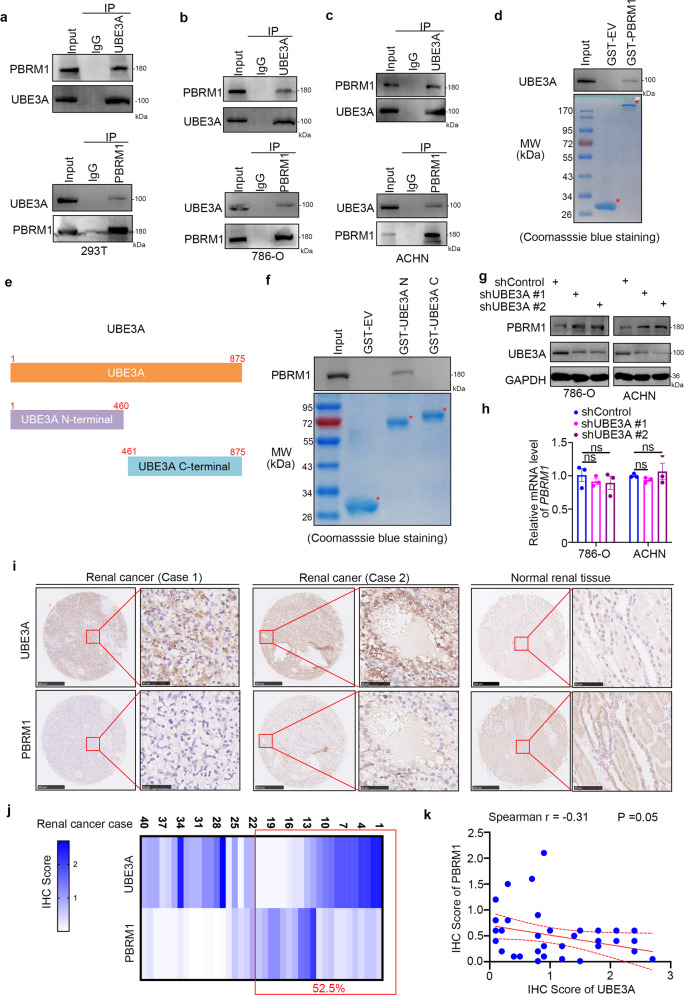
UBE3A binds PBRM1 to decrease the PBRM1 protein level in renal cancer cells. **a–c** Using the PBRM1 and UBE3A antibodies to performed the IP assay. Western blotting analysis the whole-cell lysates (WCL) of 293T (**a**), 786-O (**b**), and ACHN (**c**) cells. **d** Western blotting analysis of UBE3A proteins in 786-O whole-cell lysates pulled down by GST-EV or GST-PBRM1 recombinant proteins. Asterisks indicated the corresponding protein band of GST-EV and GST-PBRM1. **e** A schematic diagram depicting a set of GST-UBE3A recombinant protein constructs. **f** Western blotting analysis of PBRM1 proteins in 786-O whole-cell lysates pulled down by GST-EV or GST-UBE3A recombinant proteins. Asterisks indicated the corresponding protein band of GST-EV and GST-UBE3A recombinant proteins. **g,** **h** 786-O and ACHN cells were infected with indicates shRNAs for 72 h. Cells were harvested for western blotting analysis (**g**) and RT-qPCR assay (**h**). Statistical significance was determined by one-way ANOVA followed by Tukey’s multiple comparisons test. Data presented as mean ± SEM with three replicates (*n* = 3). ns not significant. **i**–**k** IHC analysis of the tissue microarray with a cohort of patients with renal cell carcinoma by using the UBE3A and PBRM1 antibodies. The typical images of IHC were shown in (**i**). Heatmap showing the IHC score of PBRM1 and UBE3A in (**j**). Correlation analysis of the IHC score of PBRM1 and UBE3A proteins in (**k**).

Since the post-translational modification of PBRM1 is poorly understood, we applied mass spectrometry analysis of PBRM1 to study how PBRM1 is regulated in cells (Supplementary Table 2, Supplementary Fig. 2a). Here, we aimed to explore whether the ubiquitination modification of PBRM1 was also presented in renal cell carcinoma cells. Therefore, we focused on finding the E3 ligase from the mass spectrometry analysis of PBRM1 (Supplementary Table 2). Numbers of proteins were identified to interact with PBRM1 (Supplementary Table 2, Supplementary Fig. 2a). Although we found that UBE3A was a far down list of the list of proteins identified to bind PBRM1 and consists of a single identified peptide fragment (Supplementary Fig. 2a, b and Supplementary Table 2). We would like to check the relationship between PBRM1 and UBE3A. The subsequent coimmunoprecipitation experiment verified that PBRM1 and UBE3A could bind to each other in 293T and renal cancer cell lines (786-O and ACHN cells) (Fig. 1a–c). Then, a GST pull-down assay indicated that UBE3A interacted with PBRM1 in vitro (Fig. 1d). Furthermore, we constructed two GST-UBE3A recombinant proteins, GST-UBE3A N-terminus (1–460) and GST-UBE3A C-terminus (461–875), to test which region of UBE3A is critical for PBRM1 binding (Fig. 1e). GST pull-down assays showed that PBRM1 is specifically bound to the N-terminal fragment of UBE3A (Fig. 1f). The results also showed that decreased UBE3A expression induced an increase in the protein level rather than the mRNA level of PBRM1 in the 786-O and ACHN cells (Fig. 1g, h). In addition, we checked the clinical relevance of PBRM1 and UBE3A in a tissue microarray of patients with renal cancer. Representative images of stained PBRM1 and UBE3A were shown in Fig. 1i. The specific IHC scores of PBRM1 and UBE3A were indicated in Fig. 1j. We found about 52.5% of patients (21/40) showed regulation of PBRM1 at the protein level by UBE3A (Fig. 1j). We also included a consolidated graph of 40 patients showing both UBE3A and PBRM1 inverse correlation in Fig. 1k. The statistical analysis indicated that there was a negative association between PBRM1 and UBE3A in this tissue microarray (Spearman correlation r = −0.31 *P* = 0.05) (Fig. 1i). Together, these findings demonstrate that UBE3A interacts with PBRM1 and UBE3A silencing increases the PBRM1 protein levels in renal cancer cells.

### UBE3A promotes PBRM1 degradation in renal cancer cells

**Fig. 2:**
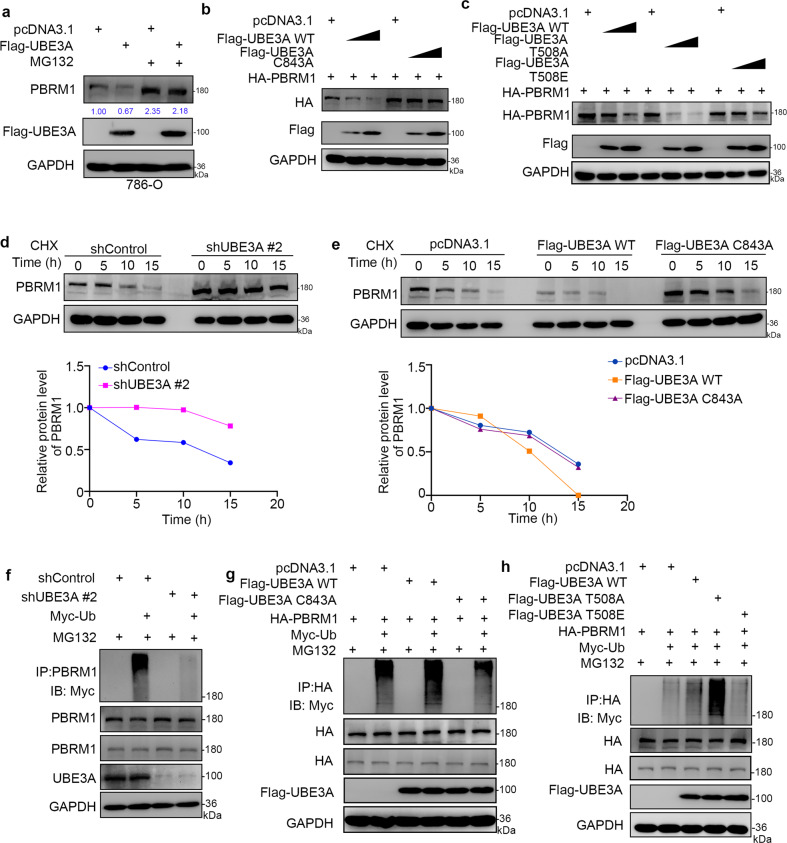
UBE3A promotes PBRM1 degradation in renal cancer cells. **a** 786-O cells were transfected with indicated plasmids for 24 h. Before harvested for western blotting analysis, cells were treated with or without 20 µM MG132 for 6 h. **b** 786-O cells were transfected with indicated plasmids. Twenty-four hours post transfection, cells were harvested for western blotting analysis. **c** 786-O cells were transfected with indicated plasmids. Twenty-four hours post transfection, cells were harvested for western blotting analysis. **d** 786-O cells were infected with indicated shRNAs. After 72 h, cells were treated with cycloheximide (CHX), and cells were collected for western blot analysis at different timepoints. **e** 786-O cells were transfected with indicated plasmids. After 24 h, cells were treated with CHX, and cells were collected for western blot analysis at different timepoints. **f** 786-O cells were infected with the indicated shRNAs. After 72 h, cells were collected for western blotting after treatment with MG132 for 8 h. **g** 786-O cells were transfected with the indicated plasmids. After 24 h, cells were collected for Western blotting after treatment with MG132 for 8 h. **h** 786-O cells were transfected with the indicated plasmids. After 24 h, cells were collected for western blotting after treatment with MG132 for 8 h.

UBE3A acts as an E3 ligase, and the substrate interaction is the fundamental step for UBE3A-mediated protein degradation [8]. We found that the knockdown of UBE3A increased the PBRM1 protein level in renal cancer cells. We also showed that knockdown of UBE3A increased the PBRM1 protein levels, but overexpression of UBE3A reduced the PBRM1 expression in 293T cells (Supplementary Fig. 2c, 2d). Meanwhile, we also showed that the protein level of UBE3A was negatively correlated with the PBRM1 protein level in the renal cancer patient specimens (Fig. 1i, k). Therefore, we hypothesized that UBE3A might promote PBRM1 degradation in renal cancer cells. To test this hypothesis, UBE3A plasmids were transfected into the 786-O cells treated with or without 26 S proteasome inhibitor MG132. Consistent with this hypothesis, ectopically overexpressed UBE3A resulted in the downregulation of PBRM1 expression, and this process could be attenuated by MG132 treatment (Fig. 2a), which indicated that UBE3A might promote PBRM1 proteasome degradation. Moreover, we found that overexpression of the reported UBE3A E3 ligase activity-decreased mutant [10] (UBE3A C843A, the cysteine at 843 mutated to glycine) made no change in the PBRM1 protein level compared with the effect of the overexpression UBE3A wild-type (UBE3A WT) construct in 786-O cells (Fig. 2b). It has been documented that phosphorylation at threonine 508 (T508) by PKA enhanced the E3 ligase activity of UBE3A, and dephosphorylation of UBE3A at T508 enhances substrate degradation [11]. Similarly, we found that ectopic overexpression of the UBE3A-T508A (the threonine at 508 mutated to glycine) mutant, which mimics dephosphorylated UBE3A, decreased more PBRM1 levels, but the UBE3A-T508E (the threonine at 508 mutated to glutamic acid) mutant, which mimics phosphorylated UBE3A, had little effect on PBRM1 compared to UBE3A WT (Fig. 2c). Then, we showed that the knockdown of UBE3A prolonged the half-life of PBRM1 (Fig. 2d). And overexpression of UBE3A WT shorten the half-life of PBRM1, but UBE3A C843A did not change the half-life of PBRM1 (Fig. 2e). Furthermore, UBE3A was silenced and Myc-Ub plasmids were transfected into the 786-O cells. The cell lysate was immunoprecipitated with PRBM1 antibodies to detect the ubiquitination level of PBRM1 in different statuses of UBE3A. We found that the knockdown of UBE3A decreased the polyubiquitination of PBRM1 in 786-O cells (Fig. 2f). In contrast, UBE3A WT overexpression enhanced the polyubiquitination modification of PBRM1 in 786-O cells (Fig. 2g, h). While, the E3 ligase activity-decreased mutants (UBE3A C843A or UBE3A-T508E) decreased the polyubiquitination modification of PBRM1 in 786-O cells (Fig. 2g, h), the E3 ligase activity-increased mutants (UBE3A-T508A) promoted the polyubiquitination modification of PBRM1 compared with UBE3A WT in 786-O cells (Fig. 2g, h). Thus, our results indicate that PBRM1 is a bonafide substrate of UBE3A and is involved in proteasomal degradation in renal cancer cells.

### DAPK3, as a binding partner, regulates PBRM1 stability in renal cancer cells

**Fig. 3:**
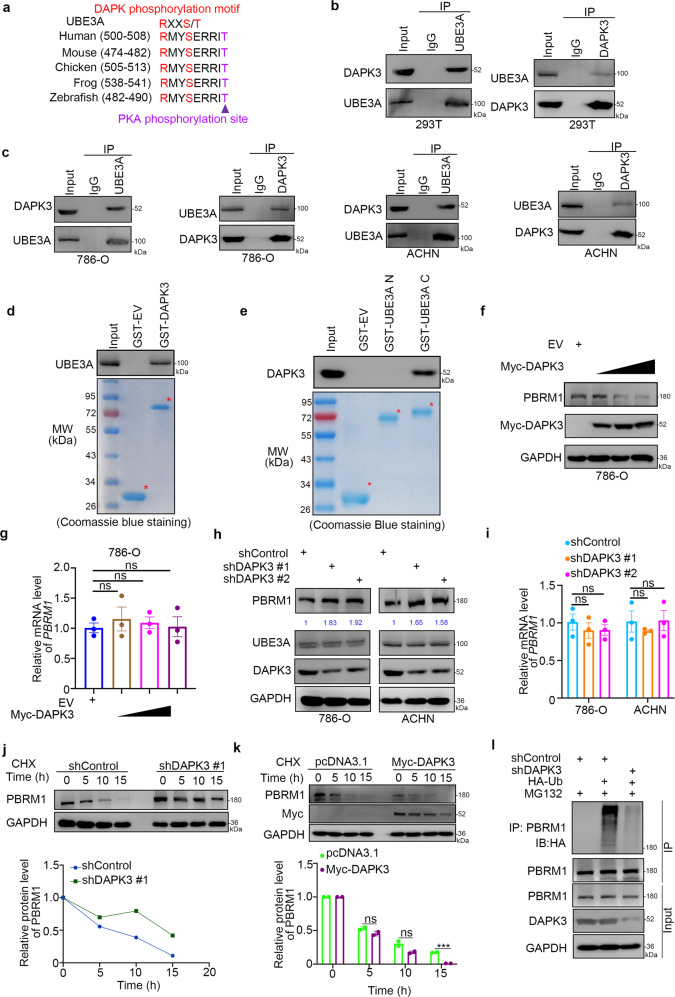
DAPK3 as a binding partner regulates PBRM1 stability in renal cancer cells. **a** A schematic diagram depicted that UBE3A contained a consensus DAPK phosphorylation motif which was adjacent to the PKA phosphorylation site. **b** Western blotting analysis the whole-cell lysates (WCL) of 293T cells. **c** Western blotting analysis the WCL 786-O and ACHN cells. **d** Western blotting analysis of UBE3A proteins in 786-O whole-cell lysates pulled down by GST-EV or GST-DAPK3 recombinant proteins. **e** Western blotting analysis of DAPK3 proteins in 786-O whole-cell lysates pulled down by GST-EV or GST-UBE3A recombinant proteins. **f**, **g** 786-O cells were transfected with indicated plasmids. Twenty-four hours post transfection, cells were harvested for Western blotting analysis (**f**) and RT-qPCR analysis (**g**). Statistical significance was determined by one-way ANOVA followed by Tukey’s multiple comparisons test. Data presented as Mean ± SEM with three replicates (*n* = 3). ns not significant. **h**, **i** 786-O cells were transfected with indicated shRNAs. Seventy-two hours post infection, cells were harvested for western blotting analysis (**h**) and RT-qPCR analysis (**i**). Statistical significance was determined by one-way ANOVA followed by Tukey’s multiple comparisons test. Data presented as Mean ± SEM with three replicates (*n* = 3). ns not significant. **j** 786-O cells were infected with indicated shRNAs. After 72 h, cells were treated with CHX, and cells were collected for western blot analysis at different timepoints. **k** 786-O cells were transfected with indicated plasmids. After 24 h, cells were treated with CHX, and cells were collected for western blot analysis at different timepoints. The GAPDH was recognized as the loading control. The protein level of PBRM1 was first normalized to loading control. The normalized values were further normalized to the values in 0 h group. Immunoblots (IB) are representative of results from two independent experiments (*n* = 2). Statistical significance was determined by multiple student’s *t*-test at the time point of 5, 10, 15 h. Data presented as Mean ± SEM with two replicates. Ns not significant; ***P < 0.001. Data presented as Mean ± SEM with two replicates. Ns not significant; ***P < 0.001. **l** 786-O cells were infected with the indicated shRNAs. After 72 h, cells were collected for western blotting after treatment with MG132 for 8 h.

Since PKA was reported to phosphorylate UBE3A and inhibit its E3 ligase activity [11]., we were curious about whether other proteins affected the function of UBE3A in renal cancer. It is worth noting that there was a DAPK3 consensus binding motif in the amino acid sequence of UBE3A, which was close to the phosphorylation site of PKA (Fig. 3a). Mass spectrometry of UBE3A was performed, and DAPK3 was also indicated as a potential binding partner of UBE3A (Supplementary Table 2 and Supplementary Fig. 2e). Thus, we wondered whether DAPK3 interacted with UBE3A and influenced the E3 ligase activity of UBE3A. At first, reciprocal coimmunoprecipitation assays confirmed the interaction between UBE3A and DAPK3 in 293T and renal cancer cell lines (786-O and ACHN cells) (Fig. 3b, c). The in vitro binding of UBE3A and DAPK3 was verified via a GST pull-down assay (Fig. 3d). In addition, we also found that DAPK3 interacted with the C-terminal fragment of UBE3A (Fig. 3e). Although UBE3A has many substrates [12–15], which may include PBRM1 but not exclusively. Here, we examined the protein level of PBRM1 to evaluate whether DAPK3 affected the activity of UBE3A. Interestingly, DAPK3 overexpression was found to decrease the protein level but not the mRNA level of PBRM1 in 786-O cells (Fig. 3f, g). In contrast, knockdown of DAPK3 by shRNAs stabilized PBRM1 expression in both 786-O and ACHN cells (Fig. 3h, i). Consistently, DAPK3 silencing prolonged the half-life of PBRM1, while DAPK3 overexpression reduced the half-life of PBRM1 in 786-O cells (Fig. 3j, k). Furthermore, we demonstrated that DAPK3 knockdown decreased the polyubiquitination of PBRM1 in 786-O cells (Fig. 3l). Together, these data suggest that DAPK3 is a binding partner of UBE3A and critical for PBRM1 degradation in renal cancer cells.

### DAPK3 competes with PKA to regulate the E3 ligase activity of UBE3A

**Fig. 4:**
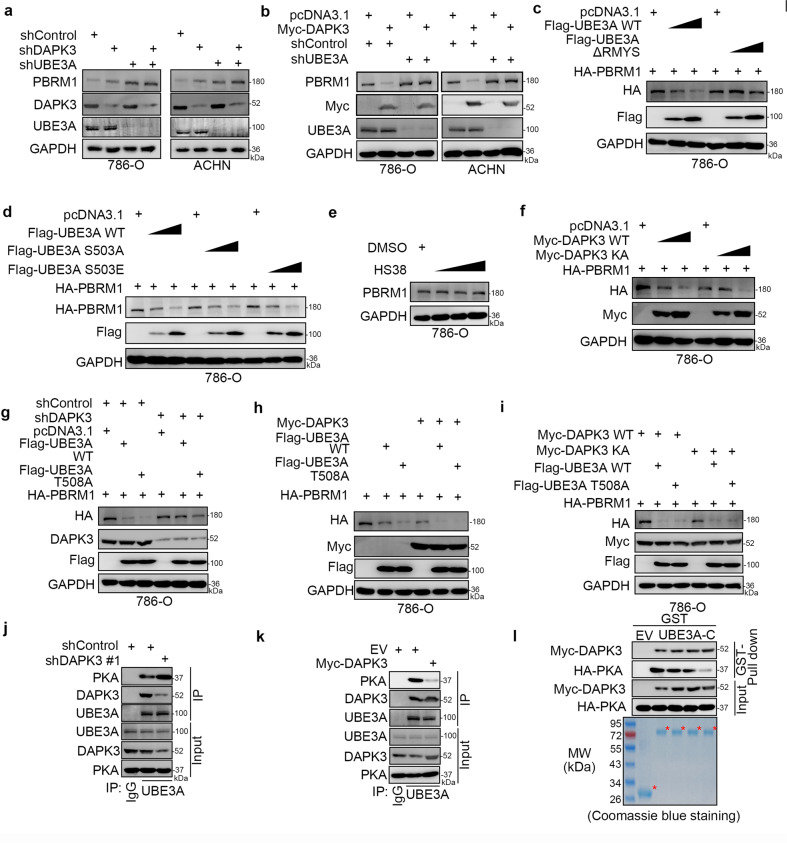
DAPK3 competes with PKA to regulate the E3 ligase activity of UBE3A. **a** 786-O and ACHN cells were infected with indicated shRNAs. Seventy-two hours post infection, cells were harvested for western blotting analysis. **b** 786-O and ACHN cells were infected with shControl and shUBE3A for 48 h. Then, 786-O and ACHN cells were transfected with indicated plasmids for another 24 h. Cells were harvested for western blotting analysis. **c** 786-O cells were transfected with indicated plasmids for 24 h. The WCL of cells were subjected to western blotting analysis. **d** 786-O cells were transfected with indicated plasmids for 24 h. The WCL of cells were subjected to western blotting analysis. **e** 786-O cells were treated with 0, 1 µM, 5 µM, and 10 µM HS38 (the DAPK3 inhibitor) for 24 h. The WCL of cells were subjected to western blotting analysis. **f** 786-O cells were transfected with indicated plasmids for 24 h. The WCL of cells were subjected to western blotting analysis. **g** 786-O cells were infected with shControl and shDAPK3 for 48 h. Then, 786-O cells were transfected with indicated plasmids for another 24 h. Cells were harvested for western blotting analysis. **h** 786-O cells were transfected with indicated plasmids for 24 h. The WCL of cells were subjected to western blotting analysis. **i** 786-O cells were transfected with indicated plasmids for 24 h. The WCL of cells were subjected to western blotting analysis. **j** 786-O were infected with shControl and shDAPK3 for 72 h. The WCL of cells were subjected to western blotting analysis. **k** 786-O cells were transfected with indicated plasmids for 24 h. The WCL of cells were subjected to western blotting analysis. **l** Western blotting analysis of in vitro expressed DAPK3 and PKA GST-pulled down by the C-terminus of UBE3A.

To explore whether DAPK3 destabilized PBRM1 through UBE3A in renal cancer cells, we performed knockdown of DAPK3 and UBE3A alone or in combination in 786-O and ACHN cells (Fig. 4a). We showed that co-knockdown of DAPK3 and UBE3A did not further increase the protein level of PBRM1 compared to the increase induced by UBE3A knockdown alone (Fig. 4a). Then, we showed that UBE3A silencing diminished the PBRM1 protein-decreasing effect induced by DAPK3 overexpression (Fig. 4b). These data indicated that UBE3A was essential for the DAPK3-induced PBRM1 decrease in renal cancer cells. Then, we further studied the underlying mechanism. Due to a DAPK3 consensus binding motif in the amino acid sequence of UBE3A (Fig. 3a), it is not surprising that overexpression of the UBE3A ^500^RMYS^503^ deletion mutant had little effect on the protein level of PBRM1 in 786-O cells (Fig. 4c). These results indicated that DAPK3 phosphorylated the serine 503 of UBE3A. We found that the UBE3A S503A (serine at 503 mutated to glycine mimicking dephosphorylated UBE3A) and S503E (serine at 503 mutated to glutamic acid mimicking phosphorylated UBE3A) mutants, manifested the same E3 ligase activity as wild-type UBE3A (Fig. 4d). Similarly, treatment with the DAPK3 inhibitor HS38 had no overt effect on the protein level of PBRM1 in 786-O cells (Fig. 4e). Moreover, overexpression of the DAPK3 kinase-dead mutant also promoted the degradation of PBRM1, similar to wild-type DAPK3 (Fig. 4f) [16]. Notably, DAPK3 knockdown weakens the degradation of PRBM1 induced by UBE3A WT or UBE3A-T508A mutant that enhanced the E3 ligase activity of UBE3A (Fig. 4g). Since UBE3A-T508E mutant decreased the E3 ligase activity of UBE3A, there is no need to use this mutant to explore the functions of DAPK3 with UBE3A. However, overexpression of DAPK3 strengthened the degradation of PRBM1 induced by UBE3A WT or T508A mutant (Fig. 4h, i), which was independent of the kinase activity of DAPK3 in renal cancer cells (Fig. 4i). Furthermore, we demonstrated that DAPK3 silencing increased the interaction between PKA and UBE3A, but DAPK3 overexpression disrupted PKA and UBE3A binding in renal cancer cells (Fig. 4j, k). We also showed that DAPK3 competed with PKA to bind with UBE3A in vitro (Fig. 4l). Therefore, our results indicate that DAPK3 competes with PKA to bind with UBE3A and enhances the degradation of PBRM1 in renal cancer cells (Fig. 4m).

### RBPJ transcriptionally increases DAPK3 expression in renal cancer cells

**Fig. 5:**
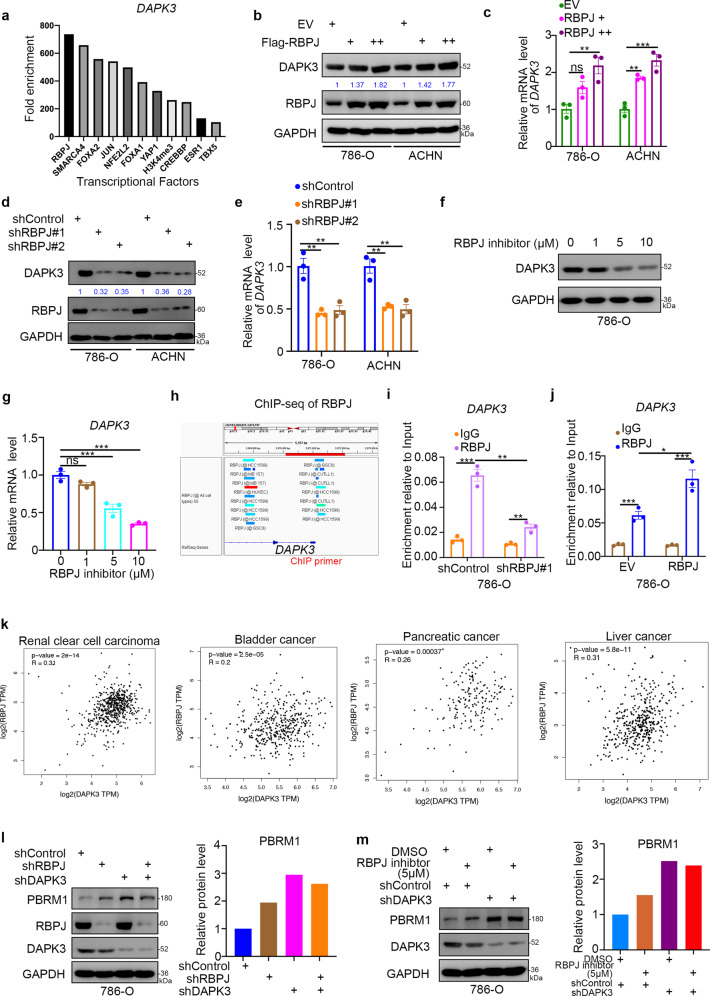
RBPJ transcriptionally increases DAPK3 expression in renal cancer cells. **a** Bioinformatic analysis of the potential binding proteins of the promoter of *DAPK3* via analysis the ChIP-seq datasets. **b**, **c** 786-O and ACHN cells were transfected with indicated plasmids for 24 h. Cells were harvested for western blotting analysis (**b**) and RT-qPCR analysis (**c**). Statistical significance was determined by one-way ANOVA followed by Tukey’s multiple comparisons test. Data presented as Mean ± SEM with three replicates (*n* = 3). Ns not significant; **P < 0.01; ***P < 0.001. **d**, **e** 786-O and ACHN cells were infected with indicated shRNAs for 72 h. Cells were harvested for western blotting analysis (**d**) and RT-qPCR analysis (**e**). Statistical significance was determined by one-way ANOVA followed by Tukey’s multiple comparisons test. Data presented as Mean ± SEM with three replicates (*n* = 3). **P < 0.01. **f**, **g** 786-O cells were treated with 0, 1 µM, 5 µM, and 10 µM the RBPJ inhibitors (the DAPK3 inhibitor) for 24 h. The WCL of cells were subjected to western blotting analysis (**f**) and RT-qPCR analysis (**g**). Statistical significance was determined by one-way ANOVA followed by Tukey’s multiple comparisons test. Data presented as Mean ± SEM with three replicates (*n* = 3). Ns not significant; ***P < 0.001. **h** the ChIP-seq of RBPJ on the promoter region of *DAPK3*. **i** 786-O cells were infected with indicated shRNAs for 72 h. Cells were harvested for ChIP-qPCR analysis by using the IgG or RBPJ antibodies. Statistical significance was determined by one-way ANOVA followed by Tukey’s multiple comparisons test. Data presented as Mean ± SEM with three replicates (*n* = 3). **P < 0.01; ***P < 0.001. **j** 786-O cells were transfected with indicated plasmids for 24 h. Cells were harvested for ChIP-qPCR analysis by using the IgG or RBPJ antibodies. Statistical significance was determined by one-way ANOVA followed by Tukey’s multiple comparisons test. Data presented as Mean ± SEM with three replicates (*n* = 3). *P < 0.05; **P < 0.01; ***P < 0.001. **k** The correlation between RBPJ and DAPK3 were analyzed by the GEPIA web tool (http://gepia.cancer-pku.cn/) in different types of cancer. **l** 786-O cells were infected with indicated shRNAs for 72 h. Cells were harvested for western blotting analysis. **m** 786-O cells were infected with indicated shRNAs for 48 h. Then, cells were treated with or without the 5 µM RBPJ inhibitor for another 24 h. Cells were harvested for western blotting analysis.

DAPK3 is a regulator of the activity of UBE3A, functioning independently of the kinase function of DAPK3. The regulation of DAPK3 might provide other clues for modulating the activity of UBE3A. A bioinformatics analysis was performed to identify the potential transcription factors of DAPK3 in cells (Fig. 5a). Among these factors, RBPJ was shown to induce the highest fold enrichment of DAPK3 (Fig. 5a). Notably, overexpressed RBPJ increased DAPK3 expression in 786-O and ACHN cells (Fig. 5b, c). However, knocking down RBPJ downregulated the protein and mRNA levels of DAPK3 in renal cancer cells (Fig. 5d, e). In addition, treatment with a serial concentration of RBPJ inhibitor (RIN1) [17] decreased DAPK3 gradually in 786-O cells (Fig. 5f, g). Moreover, the ChIP-seq analysis with multiple types of cells demonstrated that RBPJ could bind to the promoter region of DAPK3 in cells, as shown through a bioinformatics analysis (Fig. 5h). Then, a subsequent ChIP-qPCR assay indicated that RBPJ bound to the promoter of DAPK3 in 786-O cells (Fig. 5i, j). Furthermore, we explored the clinical relationship between RBPJ and DAPK3 and found that there was a positive correlation between RBPJ and DAPK3 in renal clear cell carcinoma, bladder cancer, pancreatic cancer, and liver cancer (Fig. 5k). Taken together, these data suggest that RBPJ acts as a transcription factor of DAPK3 and regulates PBRM1 in renal cancer cells. Finally, we found that RBPJ or DAPK3 knockdown alone could increase PBRM1 expression, but co-knockdown of DAPK3 and RBPJ did not further increase PBRM1 expression compared with DAPK3 knockdown alone (Fig. 5l). Similarly, RBPJ inhibitor treatment led to the upregulation of PBRM1 in 786-O cells, which was diminished by the knockdown of DAPK3 (Fig. 5m). Besides, we also showed that knockdown of DAPK3 increased more PBRM1 expression than RBPJ silencing alone or treatment with RBPJ inhibitors, which indicated that the control of DAPK3 by RBPJ was incomplete.

### RBPJ/DAPK3/UBE3A/PBRM1/p21 contributes to the resistance of renal cancer cells to CDK4/6 inhibitors

**Fig. 6:**
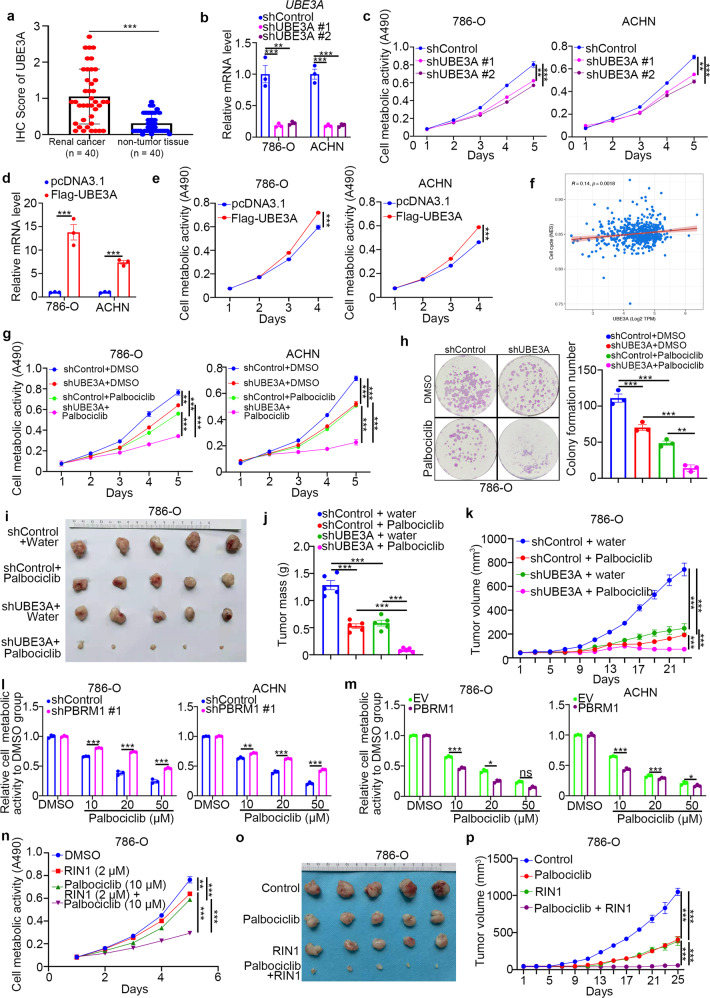
RBPJ/DAPK3/UBE3A/PBRM1/p21 contributes to the resistance of renal cancer cells to CDK4/6 inhibitors. **a** analysis of the protein levels of UBE3A in the TMA of patients with renal cell carcinoma. *N* = 40; ***P < 0.001. **b**, **c** 786-O and ACHN cells were infected with indicated shRNAs for 72 h. Cells were harvested for RT-qPCR analysis (**b**) and MTS assay (**c**). Statistical significance was determined by one-way ANOVA followed by Tukey’s multiple comparisons test. Data presented as Mean ± SEM with three replicates (*n* = 3). **P < 0.01; ***P < 0.001. **d**, **e** 786-O and ACHN cells were transfected with indicated plasmids for 24 h. Cells were harvested for RT-qPCR analysis (**d**) and MTS assay (**e**). Statistical significance was determined by Student’s *t*-test. Data presented as Mean ± SEM with three replicates (*n* = 3). ***P < 0.001. **f** The bioinformatics analysis indicated that UBE3A is positively correlate with cell cycle in renal cancer specimens through analyzing the TCGA dataset. **g**, **h** 786-O and ACHN cells were infected with indicated shRNAs for 72 h. Cells were harvested for the MTS assay (**g**) and colony formation assay (**h**) treated with or without palbociclib (20 µM). Statistical significance was determined by one-way ANOVA followed by Tukey’s multiple comparisons test. Data presented as Mean ± SEM with three replicates (*n* = 3). **P < 0.01; ***P < 0.001. **i–k** 786-O cells were infected with indicated shRNAs for 72 h. After puromycin selection, cells were harvested and injected subcutaneously into nude mice for xenografts assay and treated with or without palbociclib. The image of tumor was shown in panel **i**. The tumor mass was demonstrated in panel **j**. The tumor growth curve was indicated in panel **k**. Statistical significance was determined by one-way ANOVA followed by Tukey’s multiple comparisons test. Data presented as Mean ± SEM with five replicates (*n* = 5). **P < 0.01; ***P < 0.001. **l** 786-O and ACHN cells were infected with indicated shRNAs for 72 h. Cells were collected for the MTS assay after treated with indicated concentration of palbociclib for another 24 h. Statistical significance was determined by Student’s *t*-test. Data presented as Mean ± SEM with three replicates (*n* = 3). **P < 0.01; ***P < 0.001. **m** 786-O and ACHN cells were transfected with indicated plasmids for 24 h. Cells were collected for the MTS assay after treated with indicated concentration of palbociclib for another 24 h. Statistical significance was determined by Student’s *t*-test. Data presented as Mean ± SEM with three replicates (*n* = 3). Ns not significant; *P < 0.05; ***P < 0.001. **n–p** 786-O cells were treated with indicated small inhibitors. Cells were subjected to MTS assay (**n**) and Xenografts assay (**o**, **p**). Statistical significance was determined by one-way ANOVA followed by Tukey’s multiple comparisons test. For MTS assay, data presented as Mean ± SEM with three replicates (*n* = 3). For xenografts assay, data presented as Mean ± SEM with five replicates (*n* = 5). **P < 0.01; ***P < 0.001.

Since the cancer-related role of UBE3A in RCC is still unclear, we first analyzed the protein levels of UBE3A in the tissue microarray described above. We found that there were higher expression levels of UBE3A in renal cancer tissues than in adjacent nontumor tissues (Fig. 6a). Knockdown of UBE3A by specific shRNAs decreased cell proliferation in both 786-O and ACHN cells (Fig. 6b, c), but overexpression of UBE3A through ectopic Flag-UBE3A transfection upregulated renal cancer cell growth ability (Fig. 6d, e). Interestingly, we demonstrated that UBE3A silencing dampened the G1- to S-phase transition in 786-O cells (Supplementary Fig. 3a). In contrast, UBE3A overexpression induced G1- to S-phase progression in 786-O cells (Supplementary Fig. 3b). We also showed that UBE3A was involved in regulating the cell cycle process through analysis of the TCGA dataset (Fig. 6f). It has been reported that UBE3A/E6AP regulates the protein level of cell-cycle-related proteins, such as p27, PML, and p53, in high-risk human papillomaviruses-related cancer [12–15]. Then, we checked whether UBE3A modulated the sensitivity of cell-cycle-related antitumor small molecules, mainly cyclin-dependent kinases, in renal cancer cells (Supplementary Fig. 3c). Notably, the IC50 ratio of CDK4/6 inhibitors (palbociclib) between the control group and UBE3A changed the most among cyclin-dependent kinases inhibitors in 786-O cells (Supplementary Fig. 3c). CDK4/6 inhibitors were approved by the FDA for the treatment of breast cancer and entered a phase II study of various types of malignant cancer [18, 19]. An in vitro study in which MTS assay and colony formation analysis were performed also indicated that knockdown of UBE3A sensitized renal cancer cells to palbociclib (Fig. 6g, h). Moreover, we demonstrated that UBE3A knockdown increased the antitumor efficiency of the CDK4/6 inhibitors in mice (Fig. 6i–k).

p21, one of the downstream target genes of p53 [20, 21], contributes to the resistance to CDK4/6 inhibitors in cancer cells [22]. Interestingly, we noticed that the combination of shUBE3A and palbociclib was more pronounced in ACHN (wild-type p53) compared to 786-O (p53 inactive) [23] (Fig. 6g), which suggested that p53 status might partially determine the effect of UBE3A-induced CDK4/6 inhibitors resistance. It has been reported that PBRM1 directly regulates p21 expression or promotes p21 expression through p53 [21, 24]. As the substrate of UBE3A, we would like to check whether UBE3A regulated the sensitivity of CDK4/6 inhibitors through PBRM1/p21 axis. We silenced PBRM1 with a PBMR1 specific shRNA and treated cells with a serial concentration of palbociclib for 24 h (Fig. 6l, Supplementary Fig. 4a). The MTS assays showed that knockdown of PBRM1 decreased the antitumor effect of palbociclib in both 786-O and ACHN cells (Fig. 6l). In contrast, overexpressed PBRM1 sensitized renal cancer cells to CDK4/6 inhibitors (Fig. 6m). Besides, we also showed that PBRM1 silencing promoted renal cancer cells proliferation, which overexpressed PBRM1 inhibited the cancer cells' growth in vitro (Supplementary Fig. 4b, c). Then, we showed that knockdown of p21 resulted in palbociclib resistance in 786-O and ACHN cells (Supplementary Fig. 5a, b). And silencing p21 modestly attenuated the PBRM1-induced sensitivity to CDK4/6 inhibitors in 786-O cells (Supplementary Fig. 5c–f). The possible reason might be that although 786-O cells harbor mutant p53, palbociclib decreased the pRb-S795 levels but slightly increased the p21 expression level in 786-O cells (Supplementary Fig. 5g), as reported in the literature [25]. Then, we showed that PBRM1 determined the resistance of CDK4/6 inhibitors and led to changes in UBE3A expression in 786-O cells (Supplementary Fig. 5h–k). As the RBPJ/DAPK3 axis regulates PBRM1 expression through UBE3A, we found that RIN1 manifested synergistic anticancer effects when combined with palbociclib in cells and in mice (Fig. 6n–p). In summary, our results demonstrate that the RBPJ/DAPK3/UBE3A/PBRM1/p21 signaling pathway regulated the sensitivity of renal cancer cells to CDK4/6 inhibitors.

## Discussion

The role of UBE3A in malignant tumors has been extensively studied [8]. UBE3A degrades the tumor suppressor protein p53 to promote human papillomavirus (HPV)-associated cancer progression [26]. In addition, UBE3A is hyperactivated in Epstein-Barr virus-associated Burkitt’s lymphoma and is involved in degrading the tumor repressor promyelocytic leukemia protein (PML) [14]. Moreover, UBE3A acts as an oncogenic protein that directly regulates the protein stability of p27 and clusterin in prostate cancer [27–29]. Here, we showed that genetically repressing UBE3A expression decreased the cell proliferation activity of renal cancer cells. Furthermore, our results showed that the well-known tumor suppressor protein PBRM1 was a bonafide substrate of UBE3A for degradation. Although only the mass spectrometry data of PBRM1 indicated that PBRM1 interacted with UBE3A, there was no PBRM1 in the mass spectrum data of UBE3A. The reason might be that the mass spectrometry data are very random, and not all proteins that can bind to UBE3A will appear in the mass spectrometry data of UBE3A at one time. We have confirmed the interaction of UBE3A and PBRM1 in the following co-IP and GST pull-down assay. However, the cancer-related role of UBE3A in RCC remains unclear, and more investigation should be performed in the future.

Next-generation sequencing revealed that PBRM1 is the second most highly mutated gene in renal cancer [30]. It has been reported that the inactivation of PBRM1 helps renal cancer cells escape p53-mediated cell growth by regulating the transcriptional activity of p53 [31]. In addition, PBRM1 coupled with p53 has been shown to increase p21 expression in malignant tumor cells [21]. The role of PBRM1 in the systemic therapy of renal cancer is unknown. The investigation into the correlation of PBRM1 mutation and the outcomes of anti-VEGF and anti-mTOR pathway treatment shows that PBRM1 mutation is associated with longer progression-free survival time in patients treated with either TKIs or mTOR inhibitors [32, 33]. Thus, PBRM1 might be a promising predictor for the targeted therapy of renal cancer and the predictive role of PBRM1 in targeted therapies of renal cancer needs to be further studied. CDK4/6 inhibitors are approved by the Food and Drug Administration (FDA) for the targeted therapy of patients with breast cancer [34, 35]. Dehong Chen et al. demonstrated that CDK4/6 inhibitors inhibited renal cancer cell proliferation at clinically suitable concentrations [36]. Here, we found that the UBE3A/PBRM1/p21 axis was involved in modulating the sensitivity of CDK4/6 inhibitors in RCC. UBE3A/E6AP was reported to regulate the protein level of p53 in high-risk human papillomaviruses-related cancer [18]. Given that p53 is a primary regulator of p21 [25], and is known to be upregulated in ~36% of RCC [37]. We noticed that the combination of shUBE3A and palbociclib was more pronounced in ACHN (wild-type p53) compared to 786-O (p53 inactive) [23] (Fig. 6g), which suggested that p53 status might partially determine the effect of UBE3A-induced CDK4/6 inhibitors resistance. Besides, it has been reported that PBRM1 directly regulates p21 expression or promotes p21 expression through p53 [21, 24]. Thus, UBE3A/PBRM1/p21 axis might regulate the sensitivity of CDK4/6 inhibitors in p53 dependent or independent manner. Moreover, we showed that RBPJ/DAPK3 axis regulated the activity of UBE3A, and RBPJ inhibitors enhanced the antitumor effect of CDK4/6 inhibitors by regulating the DAPK3/UBE3A axis in RCC, which provides a clue for targeted therapy of RCC.

**Fig. 7:**
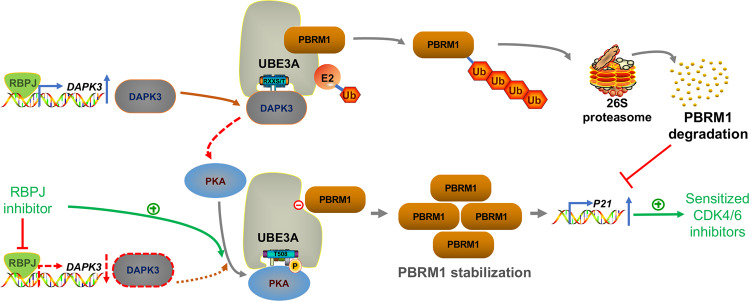
A hypothesis model depicted that PKA phosphorylated UBE3A to prevent UBE3A degrading PBRM1. DAPK3 competed with PKA to bind with UBE3A and enhance the PBRM1 degradation in renal cancer cells. PBPJ transcriptionally regulated DAPK3 expression and then promoted UBE3A-mediated degradation of PBRM1. Then, PBRM1 increased the p21 expression and sensitized renal cancer cells to CDK4/6 inhibitors. In combination with RBPJ inhibitors, CDK4/6 inhibitors synergistically enhanced renal cancer cells.

In summary, we demonstrated that UBE3A functions as a newly found E3 ligase of PBRM1 in renal cancer cells. DAPK3 competed with PKA to prevent the phosphorylation of UBE3A and enhanced its E3 ligase activity. PBPJ transcriptionally regulated DAPK3 expression and then promoted UBE3A-mediated degradation of PBRM1. Moreover, our data suggest that the RBPJ/DAPK3/UBE3A/PBRM1/p21 axis modulated the sensitivity of renal cancer cells to CDK4/6 inhibitors. In combination with RBPJ inhibitors, CDK4/6 inhibitors synergistically enhanced renal cancer cells (Fig. 7). Therefore, we not only revealed a novel RBPJ/DAPK3/UBE3A/PBRM1/p21 axis but also identified a combination strategy for overcoming the resistance of renal cancer cells to CDK4/6 inhibitors.

## Supplementary information

Supplementary information

Table S2

declaration-of-competing interests

authorship change form

Original western blot

agreement from all author

aj-checklist

## Data Availability

The datasets used and/or analyzed during the current study are available from the corresponding authors (jinxinxy2@csu.edu.cn) on reasonable request.
