# Supplementary material for: The RBPJ/DAPK3/UBE3A signaling axis induces PBRM1 degradation to modulate the sensitivity of renal cell carcinoma to CDK4/6 inhibitors
Source: Cell Death Dis. 2022 Apr 2;13(4):295. doi: 10.1038/s41419-022-04760-6 (PMC8976838; doi:10.1038/s41419-022-04760-6)
Supplement: Supplementary file 1 — Supplementary information [file 41419_2022_4760_MOESM1_ESM.docx]

**The RBPJ/DAPK3/UBE3A signaling axis induces PBRM1 degradation to modulate the sensitivity of renal cell carcinoma to CDK4/6 inhibitors**

Wentao Liu, Bin Zhang, Dan Zhang, Feng Guo, Kun Ye, Liang Zhu, Xin Jin

**Supplementary Material and Methods**

**Glutathione S-transferase pull-down assay**

Cells were lysed with 1 × RIPA lysis buffer (P0013B, Beyotime, shanghai, China) for 30 minutes at 4 ℃. Glutathione S-transferase (GST) fusion proteins were immobilized on BeyoMag™ Anti-GST Magnetic Beads (P2138, Beyotime, shanghai, China). After washed with 1 × RIPA lysis buffer, the beads were incubated with cell lysates for 4 hours. The beads were then washed four times with 1 × RIPA lysis buffer and resuspended in loading buffer. The bound proteins were subjected to SDS/PAGE and Western blotting.

**Quantitative reverse transcription PCR (RT-qPCR) and Chromatin immunoprecipitatio- qPCR (ChIP-qPCR)**

For RT-qPCR, total RNA was extracted by Eastep Super Total RNA extraction kit (LS1040, Promega, USA). The cDNA was synthesized by cDNA reverse transcription kit (4368814, ThermoFisher, USA). At the end of each PCR cycle, the amplification products were analyzed by melting curves. GAPDH was used as internal control, and the gene expression was quantitated by 2 -^△△^CT method. The PCR primer sequences were as follows: *PBRM1* forward primer 5，- TCAGGGCACAGTGACTGAAG - 3，, reverse primer 5，- TATCTTTGGCCATTGCATGA - 3; *DAPK3* forward primer 5，- TGCACGACATCTTCGAGAAC - 3，, reverse primer 5，- GTTCTTGTCCAGCAGCATGA - 3，; *UBE3A* forward primer 5，- CCCTGATGATGTGTCTGTGG - 3，, reverse primer 5，- GGCAAAGCCATTTCCAGATA - 3，*GAPDH* forward primer 5，- CCAGAACATCATCCCTGCCT - 3， , reverse primer 5，- CCTGCTTCACCACCTTCTTG - 3，. For ChIP-qPCR, the procedure was described previously ^1^. Cell lysates were subjected to immunoprecipitaion with anti-RBPJ or anti-IgG antibodies. The ChIP-qPCR primers were provided as follows: *DAPK3* forward primer 5，- GATTCTCTCCCTCCCTCGTC - 3，, reverse primer 5，- GCTGCGGAGGGTAAAGAGC- 3，

**Cell proliferation analysis**

For colony formation assay, after cultured for 24h, cells were digested with trypsin and counted. Cells were resuspended in the complete medium, and the cell density was adjusted to 1×10^3^ /mL. Add 2mL complete medium and 100μL cell suspension to each well in a 6-well plate. Each cell sample was inoculated with 3 duplicate wells. Cells were cultured under standard conditions, and the medium was changed every 3 days to observe the formation of clones. When the cells formed visible clones (about 2 weeks), the culture was terminated. The medium was discarded, washed twice with PBS, fixed at room temperature for 30min with 4% PFA, and stained for 30min with Gimsa. The staining solution was slowly washed off with PBS and dried in the fume hood. Each cell under an inverted microscope was counted according to the same criteria for clones (more than 30 cells) and photographed.

For MTS assay, renal cancer cells (1× 10^4^ cells/well) were seeded in 96-well plates and incubated for 24h. Then, the medium was replaced with fresh medium added with MTS reagent (ab197010, Abcam), and the absorbance of each well at 490 nm.

**Public datasets for data mining and bioinformatics analysis**

**Upstream targets of the key gene**

Upstream targets of the key gene were determined by ChIP-seq and correlation analysis. Binding site in the targeted gene promoter of the key protein or methylation were obtained from ChIP-Atlas database (https://chip-atlas.org/). Correlation analysis between gene/protein and targeted genes was applied to further validate the ChIP-seq results.

**Statistical analysis and visualization**

Microsoft R Open v4.0.2 was used for data mining, bioinformatics analysis and visualization in transcriptomics data. IGV v2.9.0 was used for analysis and visualization of ChIP-seq data.

**Correlation analysis**

The GEPIA web tool (http://gepia.cancer-pku.cn/) was used to analyze the correlation between RBPJ and DAPK3.

For analyzing the correlation between cell cycle and UBE3A, renal cancer patients were first divided into two groups according to the median expression level of the key gene. Then, differential expression analysis was applied between the high and low expression groups. Input genes for GSEA were sorted by their logFC values. Signaling pathways activated or suppressed by the key gene were decided by the normalized enrichment score (NES) value derived from GSEA. Correlation analysis was performed between expression values of key gene and NES of signaling pathways.

**Liquid chromatography-tandem mass spectrometry/mass spectrometry analysis**

For the mass spectrometry analysis of PBRM1, the cell lysates of 293T cells were collected and immunoprecipitated with IgG antibodies or PBRM1 and protein A+G agarose beads (#P2012, Beyotime, Shanghai, China) at 4 °C. The mass spectrometry analysis was conducted by SpecAlly Life Technology Co., Ltd, Wuhan, China.

Sample preparation: the beads samples obtained from immunoprecipitation experiment were washed three times with pre-cooled PBS buffer to remove the remaining detergent. Then beads samples were incubated in the reaction buffer (1% SDC/100 mM Tris-HCl, pH 8.5/10 mM TCEP/40 mM CAA) at 95 °C for 10 min for protein denaturation, cysteine reduction and alkylation. The eluates were diluted with equal volume of H2O and subjected to trypsin digestion overnight by adding 1 μg of trypsin at 37 °C. The peptide was purified using self-made SDB desalting columns. The eluate was vacuum dried and stored at -20 °C for later use.

LC-MS/MS Detection: LC-MS/MS data acquisition was carried out on a Q Exactive HF-X mass spectrometer coupled with an Easy-nLC 1200 system (both Thermo Scientific). Peptides were first loaded onto a C18 trap column and then eluted into a C18 analytical column (75 μm × 250 mm, 3 μm particle size, 100 Å pore size, Acclaim PepMap C18 column, Thermo). Mobile phase A (0.1% formic acid) and mobile phase B (80% ACN, 0.1% formic acid) were used to establish a 120 min gradient. A constant flow rate was set at 300 nL/min. For DDA mode analysis, each scan cycle consisted of one full-scan mass spectrum (R = 120 K, AGC = 3e6, max IT = 50 ms, scan range = 350–1800 m/z) followed by 20 MS/MS events (R = 60 K, AGC = 2e5, max IT = 110 ms). HCD collision energy was set to 32. Isolation window for precusor selection was set to 1.6 Da. Former target ion exclusion was set for 40 s.

Data analysis: MS raw data were analyzed with MaxQuant (V1.6.6) using the Andromeda database search algorithm. LFQ mode was checked for quantification; Variable modifications, Oxidation (M) & Acetyl (Protein N-term); Fixed modifications, Carbamidomethyl (C); Digestion, Trypsin/P; Match between runs was used for identification transfer. Search results were filtered with 1% FDR at both protein and peptide levels.

Further analysis was performed using the “proteingroups.txt” file produced by MaxQuant. First, hits to the reverse database, contaminants and proteins only identified with modified peptides were eliminated. Then the LFQ intensities were logarithmized. Samples were first grouped in triplicates and identifications were filtered for proteins having at least two valid values in at least one replicate group, respectively. After this, missing values were imputed with values representing a normal distribution around the detection limit of the mass spectrometer. To that end, mean and standard deviation of the distribution of the real intensities were determined, then a new distribution with a downshift of 1.8 standard deviations and a width of 0.25 standard deviations was created. The total matrix was imputed using these values, enabling statistical analysis. Significant changed proteins were screened out according to the fold changes of the proteins. Mean and SD of the fold changes were calculated and Mean ±1.64 SD were set as the cutoff line. Proteins with fold change out of the cutoff line were identified as significant changed proteins between groups.

The coverage unit represents how many amino acids are recognized by the primary antibody (IgG or PBRM1) for every 100 amino acids in the candidate proteins. The IgG group was considered to be the control. We choose the potential candidates which have coverage units in the PBRM1 group but not in IgG group.

For the mass spectrometry analysis of UBE3A, the cell lysates of 293T cells were collected and immunoprecipitated with IgG antibodies or UBE3A antibodies and protein A+G agarose beads (#P2012, Beyotime, Shanghai, China) at 4 °C. The IgG group was considered to be the control. Chromatography-tandem mass spectrometry/mass spectrometry (LC-MS/MS) analysis was performed using a Thermo Ultimate 3000 liquid phase combined with Q Exactive Plus high-resolution mass spectrometry at Shanghai Applied Protein Technology. The data were retrieved using MAXQUANT (v1.6.6) software (Am Klopferspitz, Martinsried, Germany) and the algorithm Andromeda. The reference database comprised the UniProt human proteome reference database. Proteins and peptides with a false discovery rate (FDR) of 1% were selected.

**Reference**

1. Zhao J, Meng Z, Xie C, Yang C, Liu Z, Wu S*, et al.* B7-H3 is regulated by BRD4 and promotes TLR4 expression in pancreatic ductal adenocarcinoma. *Int J Biochem Cell Biol* 2019, **108:** 84-91.

2. Jin X, Fang R, Fan P, Zeng L, Zhang B, Lu X*, et al.* PES1 promotes BET inhibitors resistance and cells proliferation through increasing c-Myc expression in pancreatic cancer. *J Exp Clin Cancer Res* 2019, **38**(1)**:** 463.

**
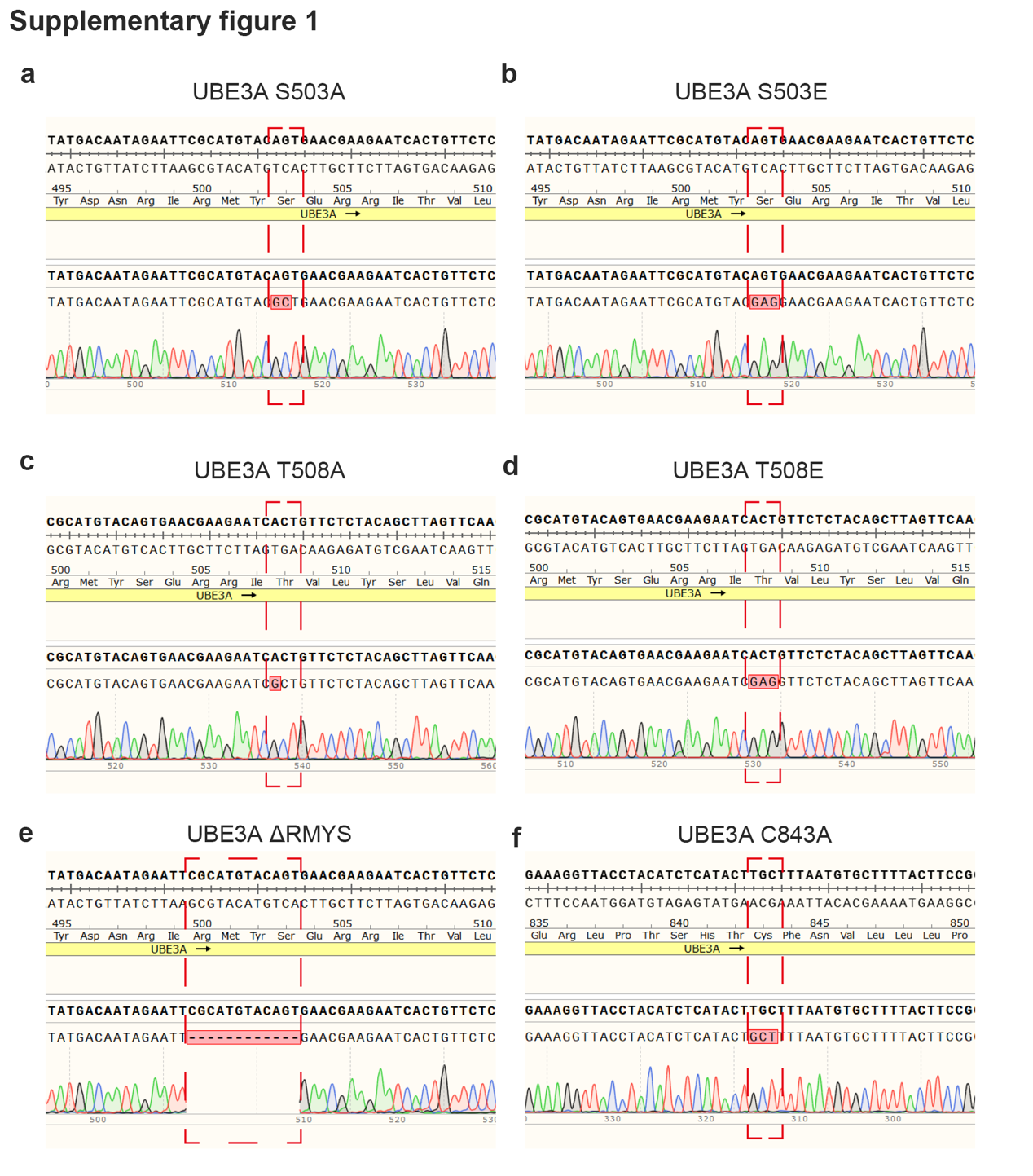
**

**Supplementary figure 1.**

**a-f,** the mutagenesis sequencing data for UBE3A.

**
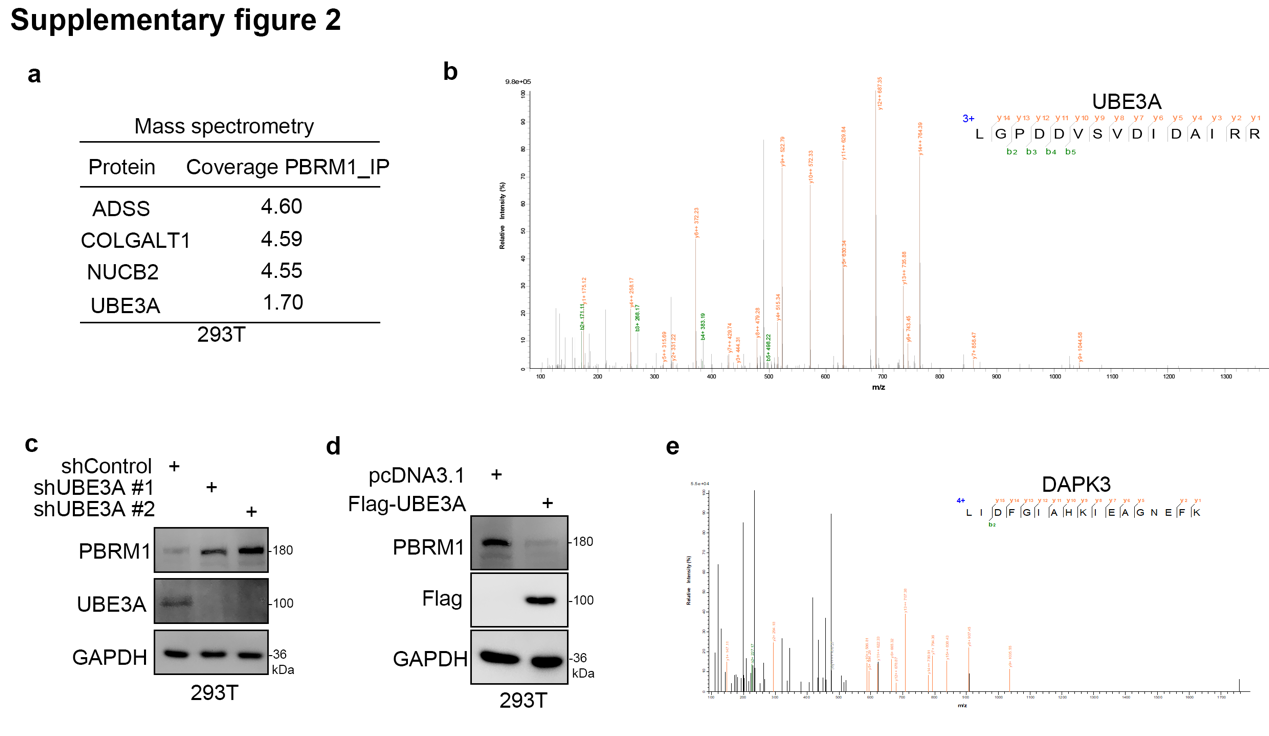
**

**Supplementary figure 2.**

**a and b,** the mass spectrometry of PBRM1 in 293T cells (a) and the peptide map of UBE3A pulled down by PBRM1 (b). **c**, 293T cells were infected with indicated shRNAs for 72 h. Cells were harvested for the Western blotting analysis. **d**. 293T cells were transfected with indicated constructs for 24 h. Cells were harvested for the Western blotting analysis. **e**, the mass spectrometry of UBE3A in 293T cells and the peptide map of DAPK3 pulled down by UBE3A.

**
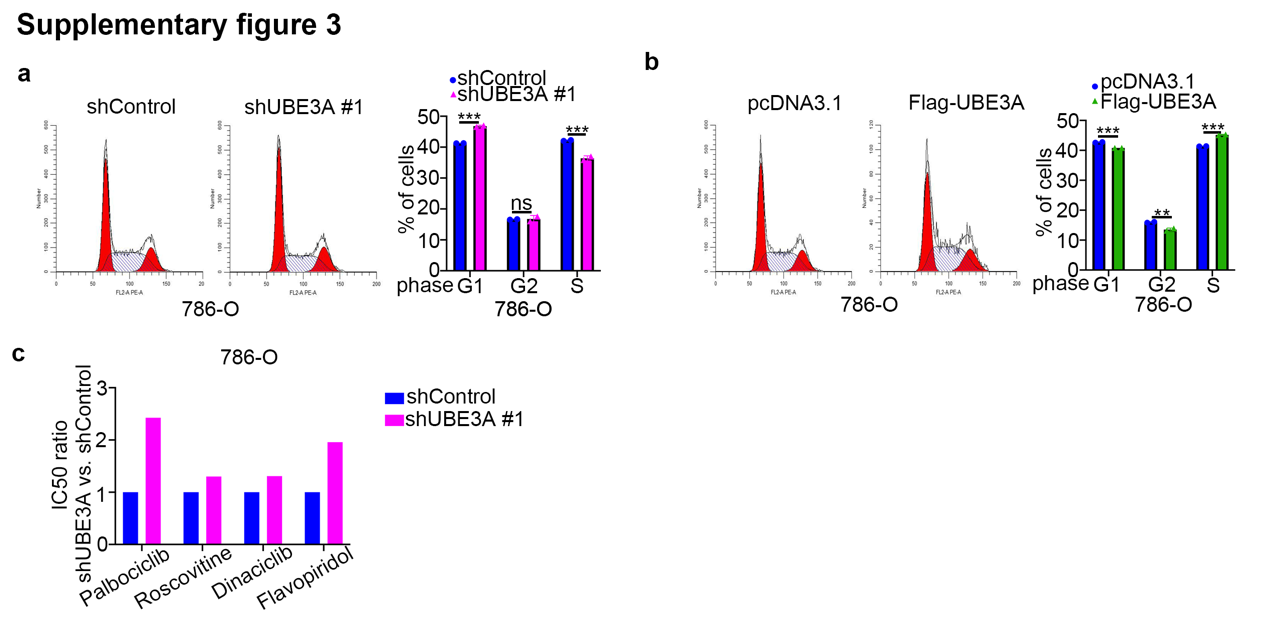
**

**Supplementary figure 3.**

**a,** 786-O cell were infected with indicated shRNAs for 72 h. Cell were harvested for the cell cycle analysis. Statistical significance was determined by Student’s *t* test. Data presented as Mean ± SEM with two replicates (n = 2). Ns, not significant; ***, P < 0.001. **b**, 786-O cells were transfected with indicated plasmids for 24 h. Cells were harvested for cell cycle analysis. Statistical significance was determined by Student’s *t* test. Data presented as Mean ± SEM with two replicates (n = 2). **, P < 0.01; ***, P < 0.001.**c**, 786-O cells were infected indicated plasmids for 72 h. These cells were treated with a serial dose of small molecules indicated in the figure and measure the IC50 values of each small molecules. The shUBE3A/shControl IC50 ratios of each small molecules were shown in the panel c.

**
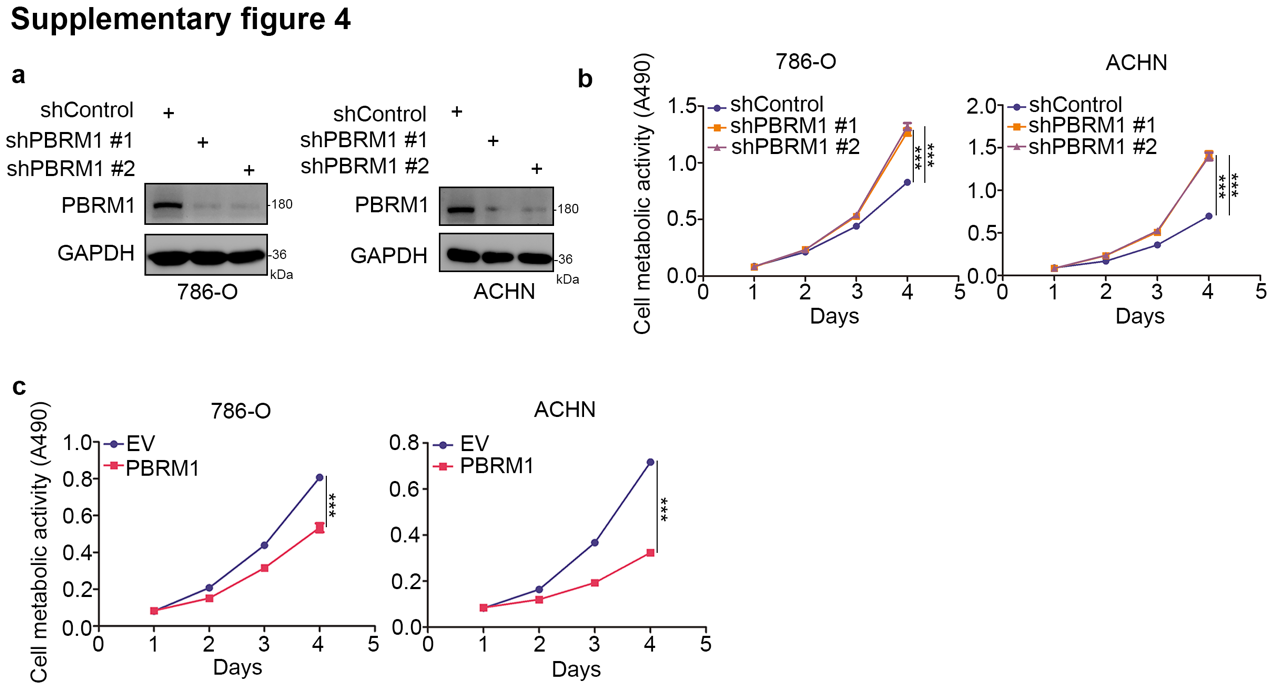
**

**Supplementary figure 4.**

**a and b,** 786-O and ACHN cells were infected with indicated shRNAs for 72 h. After puromycin selection, cells were harvested for Western blotting analysis and MTS assay. Statistical significance was determined by one-way ANOVA followed by Tukey's multiple comparisons test. Data presented as Mean ± SEM with three replicates (n = 3). ***, P < 0.001. **c**, 786-O cells were transfected with indicated plasmids for 24 h. Cells were collected for MTS assay. Statistical significance was determined by student’s *t* test. Data presented as Mean ± SEM with three replicates (n = 3). ***, P < 0.001.


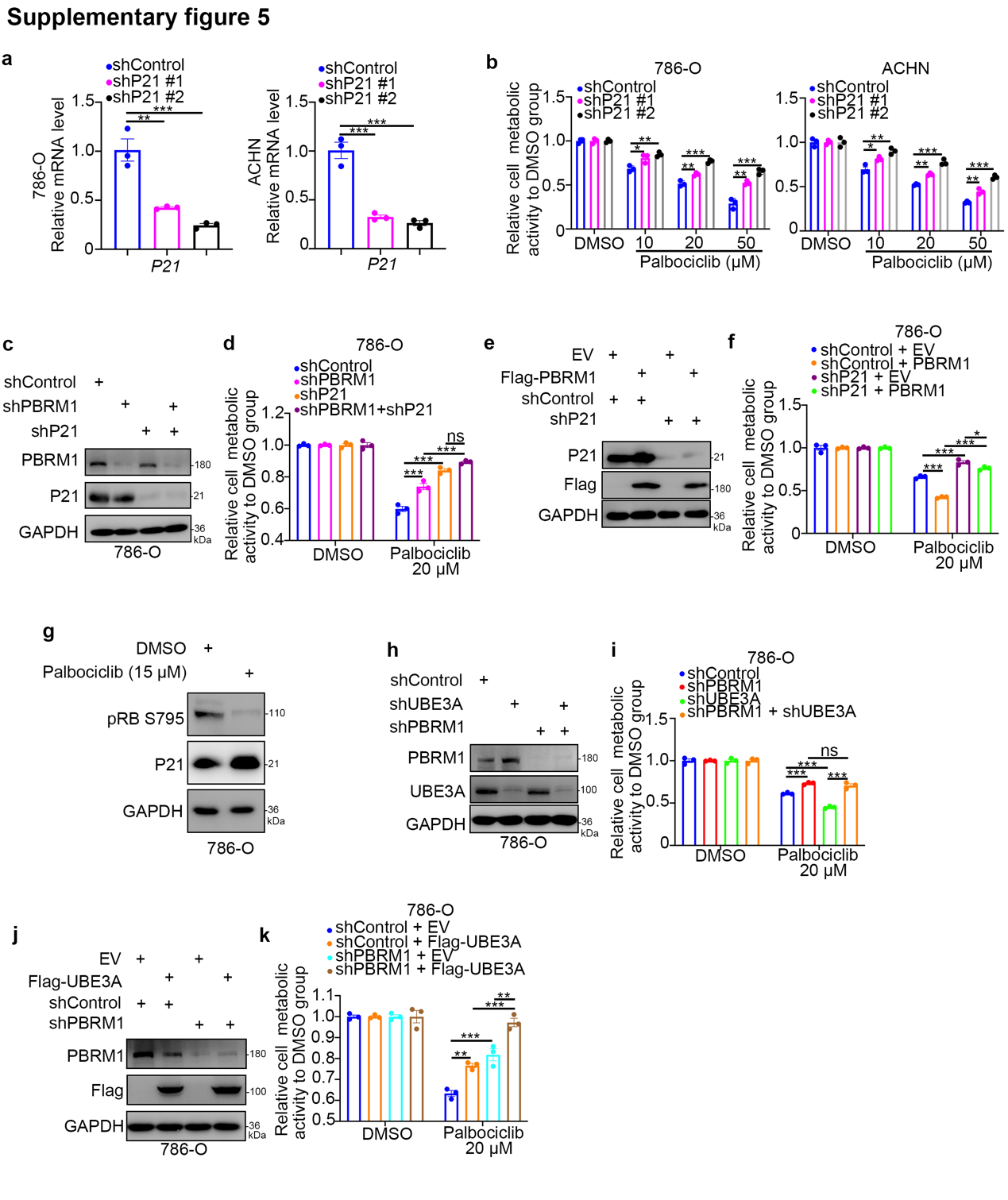


**Supplementary figure 5.**

**a and b**, 786-O and ACHN cells were infected with indicated shRNAs for 72 h. Cells were collected for RT-qPCR analysis (a) and MTS assay (b) after treated with indicated concentration of palbociclib for another 24 h. Statistical significance was determined by one-way ANOVA followed by Tukey's multiple comparisons test. Data presented as Mean ± SEM with three replicates (n = 3). *, P < 0.05; **, P < 0.01; ***, P < 0.001. **c and d,** 786-O cells were infected with indicated shRNAs for 72 h. Cells were harvested for Western blotting analysis (c) and MTS assay (d) after treated with or without 20µM palbociclib for another 24 h. Statistical significance was determined by one-way ANOVA followed by Tukey's multiple comparisons test. Data presented as Mean ± SEM with three replicates (n = 3). Ns, not significant; ***, P < 0.001. **e** **and f**, 786-O cells were infected with indicated shRNAs for 48 h. Cells were transfected with indicated plasmids for another 24 h. Then, cells were harvested for Western blotting analysis (e) and MTS assay (f) after treated with or without 20µM palbociclib for another 24 h. Statistical significance was determined by one-way ANOVA followed by Tukey's multiple comparisons test. Data presented as Mean ± SEM with three replicates (n = 3). *, P < 0.05; ***, P < 0.001. **g**, 786-O cells were treated with or without Palbociclib for 24 h. Cells were harvested for Western blotting analysis. **h and i**, 786-O cells were infected with indicated shRNAs for 72 h. Cells were harvested for Western blotting analysis (h) and MTS assay (i) after treated with or without 20µM palbociclib for another 24 h. Statistical significance was determined by one-way ANOVA followed by Tukey's multiple comparisons test. Data presented as Mean ± SEM with three replicates (n = 3). Ns, not significant; ***, P < 0.001. **j and k,** 786-O cells were infected with indicated shRNAs for 48 h. Cells were transfected with indicated plasmids for another 24 h. Then, cells were harvested for Western blotting analysis (j) and MTS assay (k) after treated with or without 20µM palbociclib for another 24 h. Statistical significance was determined by one-way ANOVA followed by Tukey's multiple comparisons test. Data presented as Mean ± SEM with three replicates (n = 3). **, P < 0.01; ***, P < 0.001.

**Table S1. Sequences of gene-specific shRNAs**

| shUBE3A-1 | 5′- CCGGCCTACATCTCATACTTGCTTTCTCGAGAAAGCAAGTATGAGATGTAGGTTTTT-3′ |
| --- | --- |
| shUBE3A-2 | 5′- CCGGCCTGATGATGTGTCTGTGGATCTCGAGATCCACAGACACATCATCAGGTTTTT-3′ |
| shDAPK3-1 | 5′- CCGGCGTCTGAAGGAGTACACCATCCTCGAGGATGGTGTACTCCTTCAGACGTTTTT-3′ |
| shDAPK3-2 | 5′- CCGGAGATTGTGAACTATGAGCCGCCTCGAGGCGGCTCATAGTTCACAATCTTTTTT-3′ |
| shPBRM1-1 | 5′- CCGGCCGGAGTCTTTGATCTACAAACTCGAGTTTGTAGATCAAAGACTCCGGTTTTT-3′ |
| shPBRM1-2 | 5′- CCGGCCGGAATGCCAGGCACTATAACTCGAGTTATAGTGCCTGGCATTCCGGTTTTTG-3′ |
| shCDKN1A-1 | 5′- CCGGCCGGCCGCGACTGTGATGCGCTACTCGAGTAGCGCATCACAGTCGCGGTTTTTTG-3′ |
| shCDKN1A-2 | 5′- CCGGCCGGGACCTGTCACTGTCTTGTACTCGAGTACAAGACAGTGACAGGTCTTTTTG-3′ |
| shRBPJ-1 | 5′- CACCGCCGTGGCTCAGGCTCCAGCGCTCGAGCGCTGGAGCCTGAGCCACGGCTTTTT-3′ |
| shRBPJ-2 | 5′-CCGGCACCCGATGGTTGTTCTGAACAACTCGAGTTGTTCAGAACAACCATCGTTTTT-3′ |
