# Supplementary material for: The RBPJ/DAPK3/UBE3A signaling axis induces PBRM1 degradation to modulate the sensitivity of renal cell carcinoma to CDK4/6 inhibitors
Source: Cell Death Dis. 2022 Apr 2;13(4):295. doi: 10.1038/s41419-022-04760-6 (PMC8976838; doi:10.1038/s41419-022-04760-6)

Figure 1

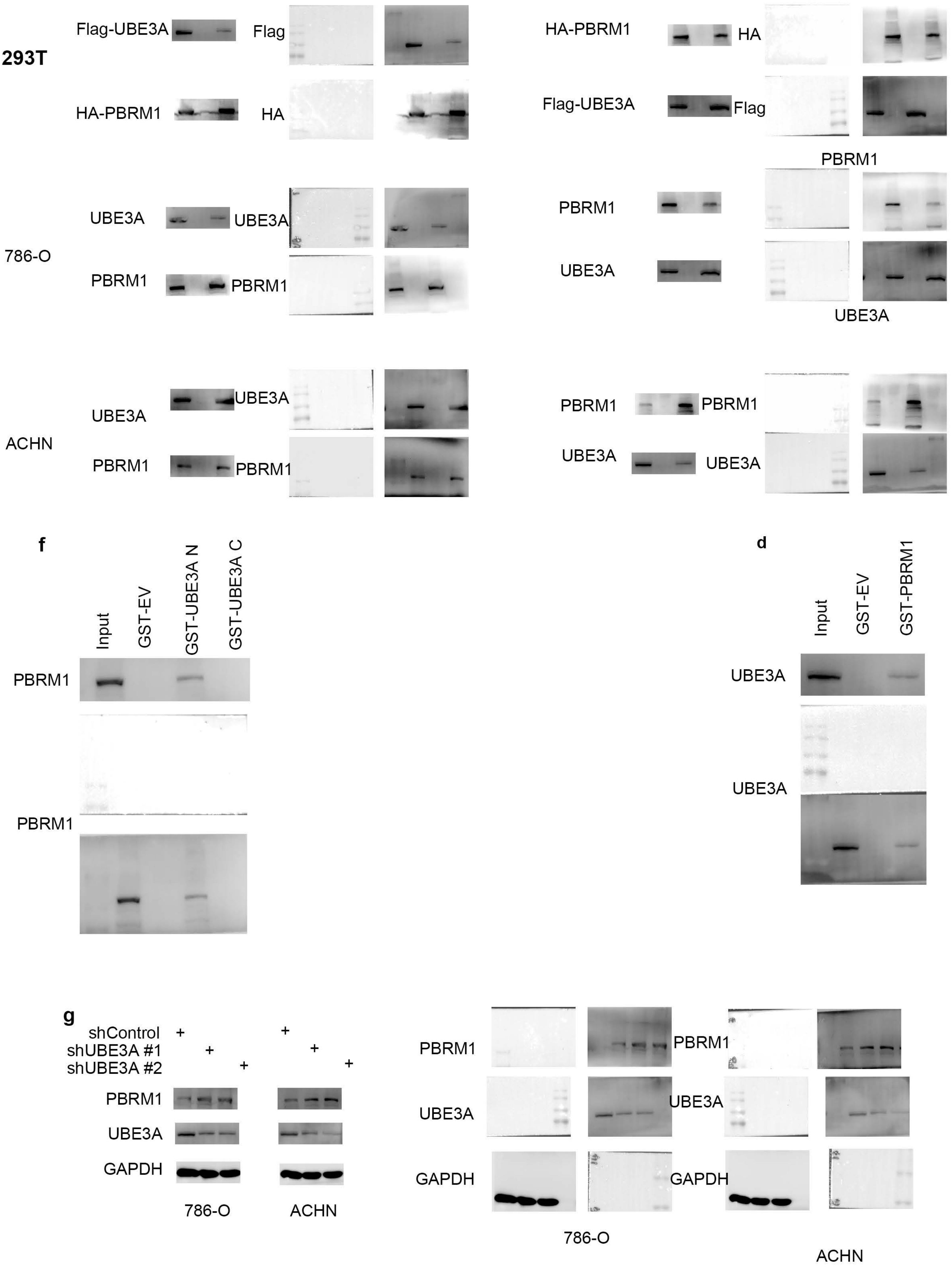

293T
Flag-UBE3A
Flag
HA-PBRM1
HA
HA-PBRM1
HA
Flag-UBE3A
Flag
PBRM1
786-O
UBE3A
UBE3A
PBRM1
PBRM1
PBRM1
UBE3A
UBE3A
ACHN
UBE3A
UBE3A
PBRM1
PBRM1
PBRM1
PBRM1
UBE3A
UBE3A
f
Input
GST-EV
GST-UBE3A N
GST-UBE3A C
PBRM1
PBRM1
d
Input
GST-EV
GST-PBRM1
UBE3A
UBE3A
g
shControl
shUBE3A #1
shUBE3A #2
PBRM1
UBE3A
GAPDH
786-O
ACHN
PBRM1
PBRM1
UBE3A
UBE3A
GAPDH
GAPDH
786-O
ACHN

Figure 2

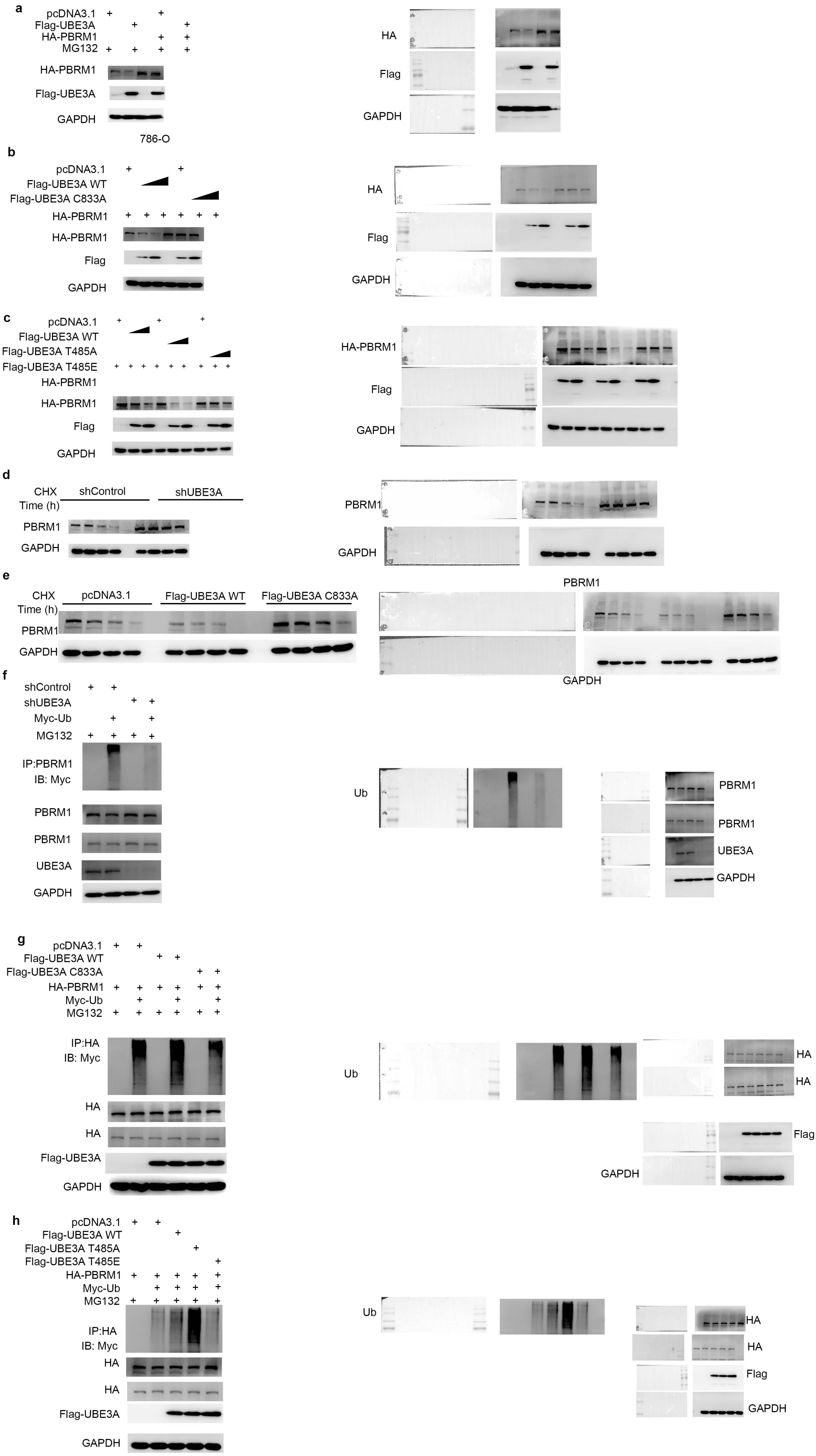
a
pcDNA3.1
Flag-UBE3A
HA-PBRM1
MG132
HA-PBRM1
Flag-UBE3A
GAPDH
786-O
HA
Flag
GAPDH
b
pcDNA3.1
Flag-UBE3A WT
Flag-UBE3A C833A
HA-PBRM1
HA-PBRM1
Flag
GAPDH
HA
Flag
GAPDH
c
pcDNA3.1
Flag-UBE3A WT
Flag-UBE3A T485A
Flag-UBE3A T485E
HA-PBRM1
HA-PBRM1
Flag
GAPDH
HA-PBRM1
Flag
GAPDH
d
CHX
Time (h)
shControl
shUBE3A
PBRM1
GAPDH
PBRM1
GAPDH
e
CHX
Time (h)
pcDNA3.1
Flag-UBE3A WT
Flag-UBE3A C833A
PBRM1
GAPDH
PBRM1
GAPDH
f
shControl
shUBE3A
Myc-Ub
MG132
IP:PBRM1
IB: Myc
PBRM1
PBRM1
UBE3A
GAPDH
Ub
PBRM1
PBRM1
UBE3A
GAPDH
g
pcDNA3.1
Flag-UBE3A WT
Flag-UBE3A C833A
HA-PBRM1
Myc-Ub
MG132
IP:HA
IB: Myc
HA
HA
Flag-UBE3A
GAPDH
Ub
HA
HA
Flag
GAPDH
h
pcDNA3.1
Flag-UBE3A WT
Flag-UBE3A T485A
Flag-UBE3A T485E
HA-PBRM1
Myc-Ub
MG132
IP:HA
IB: Myc
HA
HA
Flag-UBE3A
GAPDH
Ub
HA
HA
Flag
GAPDH

Figure 3

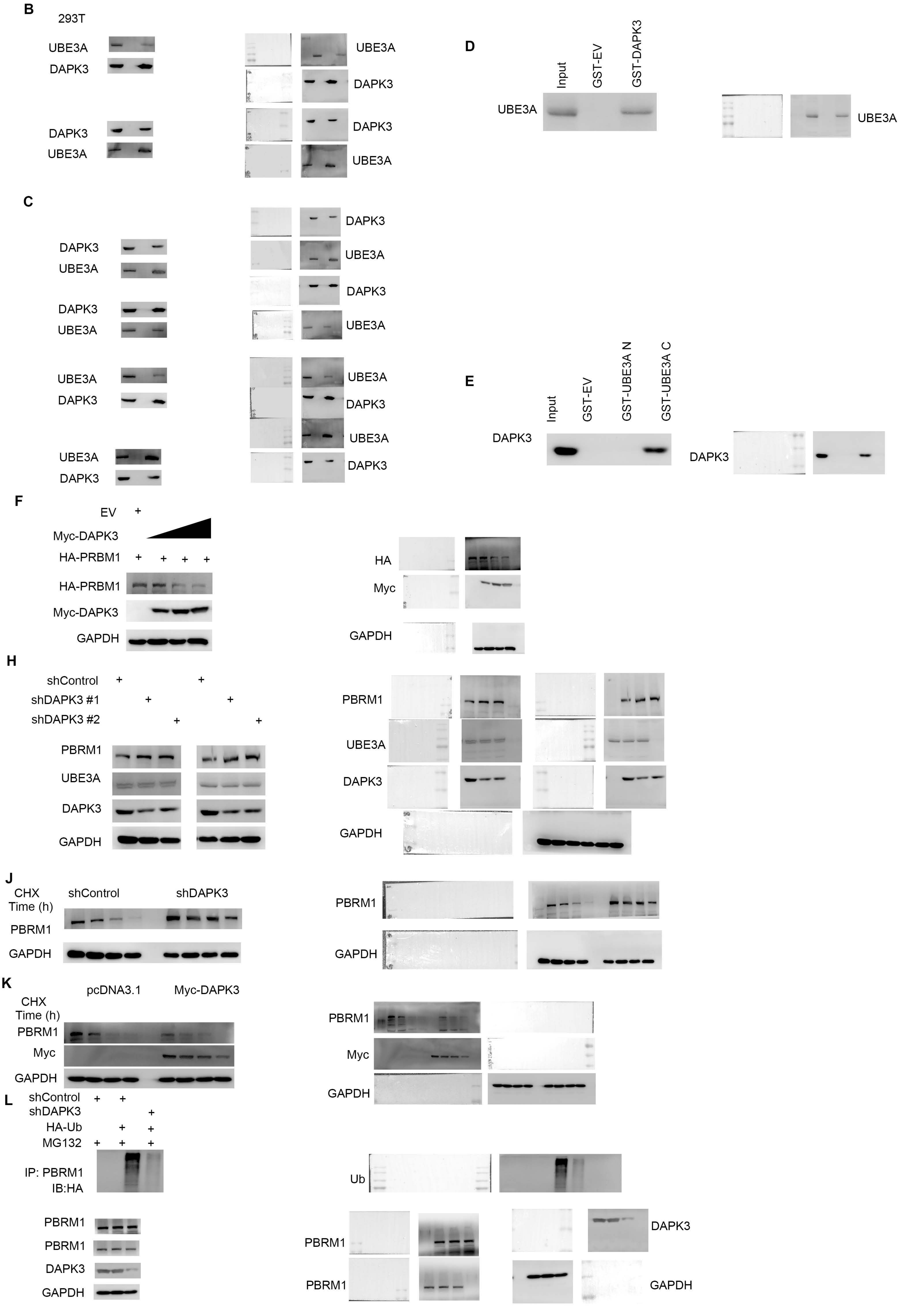
B
293T
UBE3A
DAPK3
DAPK3
UBE3A
UBE3A
DAPK3
DAPK3
UBE3A
D
Input
GST-EV
GST-DAPK3
UBE3A
UBE3A
C
DAPK3
UBE3A
DAPK3
UBE3A
UBE3A
DAPK3
UBE3A
DAPK3
DAPK3
UBE3A
DAPK3
UBE3A
UBE3A
DAPK3
UBE3A
DAPK3
E
Input
GST-EV
GST-UBE3A N
GST-UBE3A C
DAPK3
DAPK3
F
EV +
Myc-DAPK3
HA-PRBM1 + + + +
HA-PRBM1
Myc-DAPK3
GAPDH
HA
Myc
GAPDH
H
shControl + +
shDAPK3 #1 + +
shDAPK3 #2 + +
PBRM1
UBE3A
DAPK3
GAPDH
PBRM1
UBE3A
DAPK3
GAPDH
J
CHX Time (h)
shControl
shDAPK3
PBRM1
GAPDH
PBRM1
GAPDH
K
CHX Time (h)
pcDNA3.1
Myc-DAPK3
PBRM1
Myc
GAPDH
PBRM1
Myc
GAPDH
L
shControl + +
shDAPK3 +
HA-Ub + +
MG132 + + +
IP: PBRM1
IB:HA
PBRM1
PBRM1
DAPK3
GAPDH
Ub
PBRM1
PBRM1
DAPK3
GAPDH

Figure 4

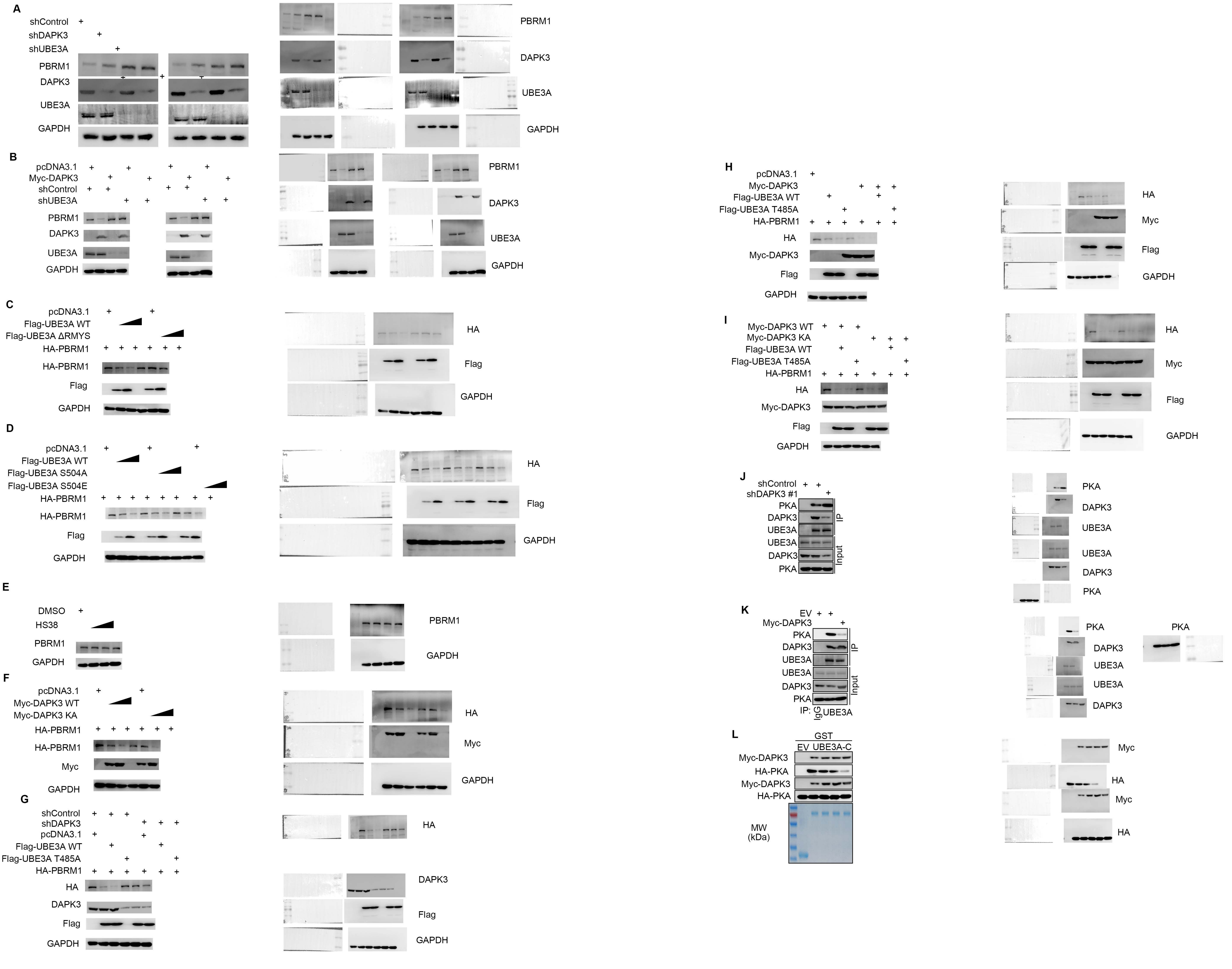
A
shControl
shDAPK3
shUBE3A
PBRM1
DAPK3
UBE3A
GAPDH
B
pcDNA3.1
Myc-DAPK3
shControl
shUBE3A
PBRM1
DAPK3
UBE3A
GAPDH
C
pcDNA3.1
Flag-UBE3A WT
Flag-UBE3A ΔRMYS
HA-PBRM1
HA-PBRM1
Flag
GAPDH
HA
Flag
GAPDH
D
pcDNA3.1
Flag-UBE3A WT
Flag-UBE3A S504A
Flag-UBE3A S504E
HA-PBRM1
HA-PBRM1
Flag
GAPDH
HA
Flag
GAPDH
E
DMSO
HS38
PBRM1
GAPDH
PBRM1
GAPDH
F
pcDNA3.1
Myc-DAPK3 WT
Myc-DAPK3 KA
HA-PBRM1
HA-PBRM1
Myc
GAPDH
HA
Myc
GAPDH
G
shControl
shDAPK3
pcDNA3.1
Flag-UBE3A WT
Flag-UBE3A T485A
HA-PBRM1
HA
DAPK3
Flag
GAPDH
HA
DAPK3
Flag
GAPDH
H
pcDNA3.1
Myc-DAPK3
Flag-UBE3A WT
Flag-UBE3A T485A
HA-PBRM1
HA
Myc-DAPK3
Flag
GAPDH
HA
Myc
Flag
GAPDH
I
Myc-DAPK3 WT
Myc-DAPK3 KA
Flag-UBE3A WT
Flag-UBE3A T485A
HA-PBRM1
HA
Myc-DAPK3
Flag
GAPDH
HA
Myc
Flag
GAPDH
J
shControl
shDAPK3 #1
PKA
DAPK3
UBE3A
UBE3A
DAPK3
PKA
IP
Input
PKA
DAPK3
UBE3A
UBE3A
DAPK3
PKA
K
EV
Myc-DAPK3
PKA
DAPK3
UBE3A
UBE3A
DAPK3
PKA
IP
Input
IP: IgG UBE3A
PKA
DAPK3
UBE3A
UBE3A
DAPK3
PKA
L
GST
EV UBE3A-C
Myc-DAPK3
HA-PKA
Myc-DAPK3
HA-PKA
MW
(kDa)
Myc
HA
Myc
HA

Figure 5

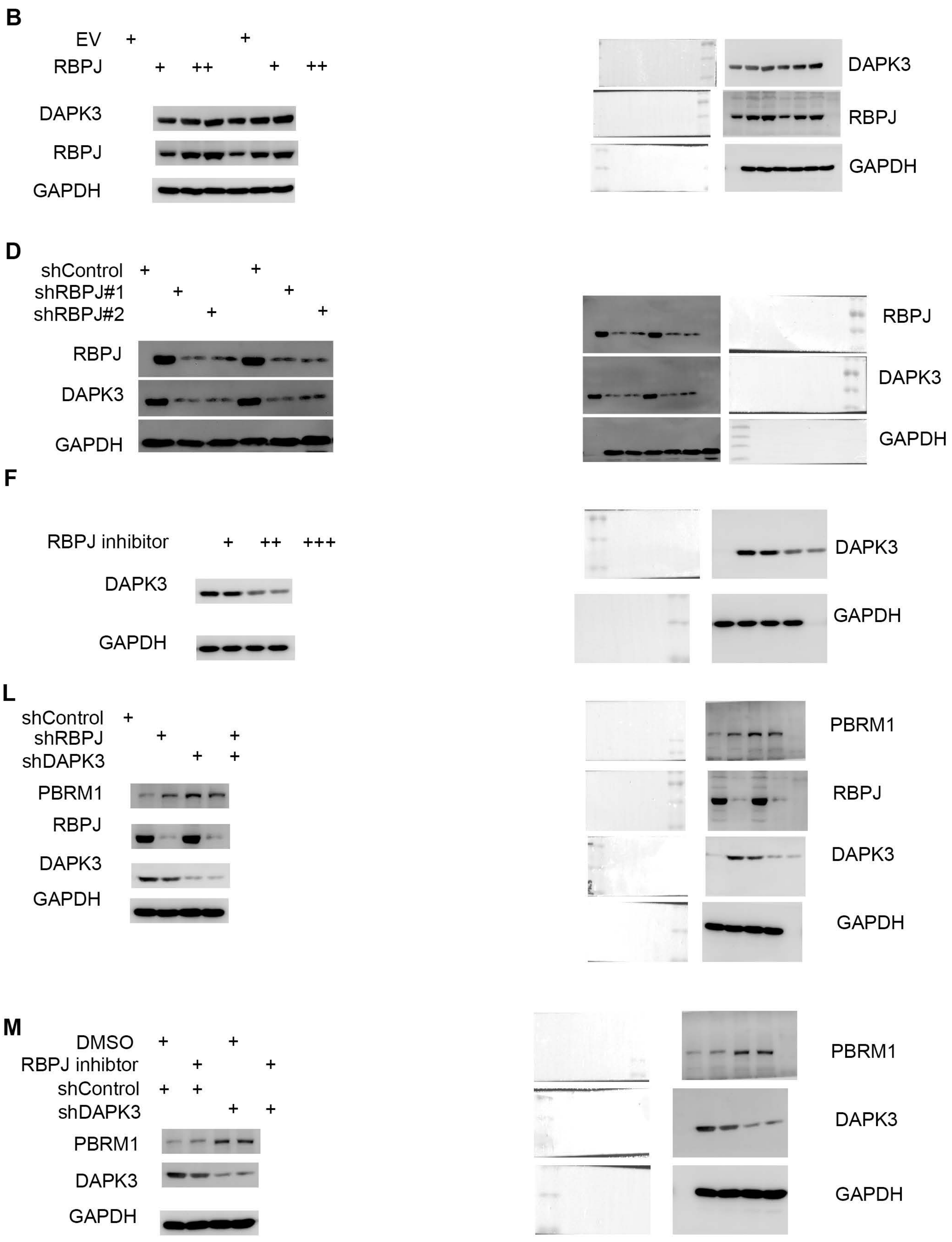
B
EV + +
RBPJ + ++ + ++
DAPK3
RBPJ
GAPDH
DAPK3
RBPJ
GAPDH
D
shControl + +
shRBPJ#1 + +
shRBPJ#2 + +
RBPJ
DAPK3
GAPDH
RBPJ
DAPK3
GAPDH
F
RBPJ inhibitor + ++ +++
DAPK3
GAPDH
DAPK3
GAPDH
L
shControl +
shRBPJ + +
shDAPK3 + +
PBRM1
RBPJ
DAPK3
GAPDH
PBRM1
RBPJ
DAPK3
GAPDH
M
DMSO + +
RBPJ inhibtor + +
shControl + +
shDAPK3 + +
PBRM1
DAPK3
GAPDH
PBRM1
DAPK3
GAPDH

Supplementary figure 2

c

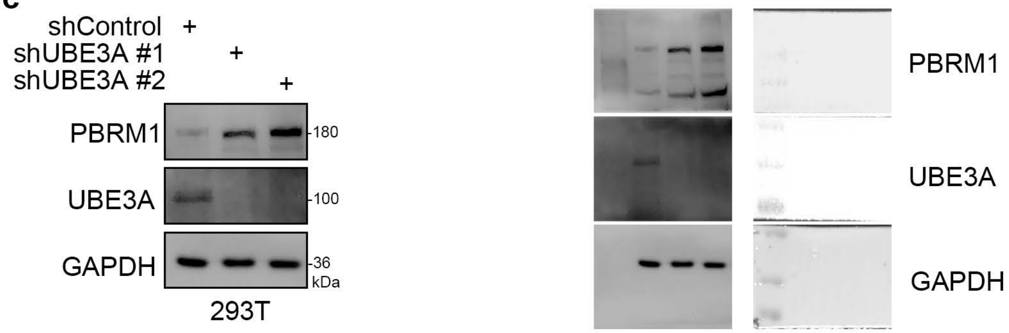

d

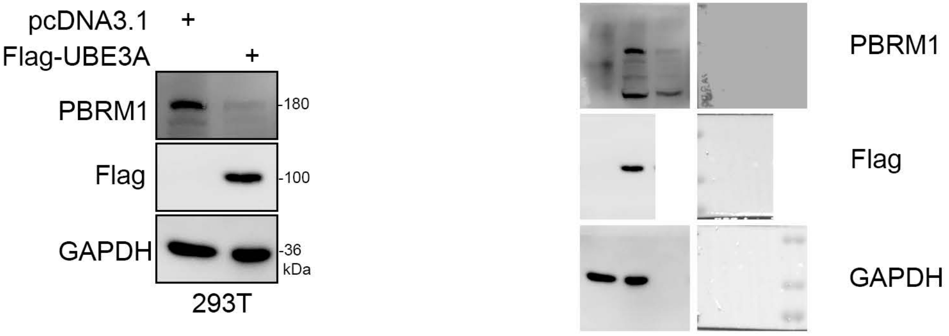

Supplementary figure 4

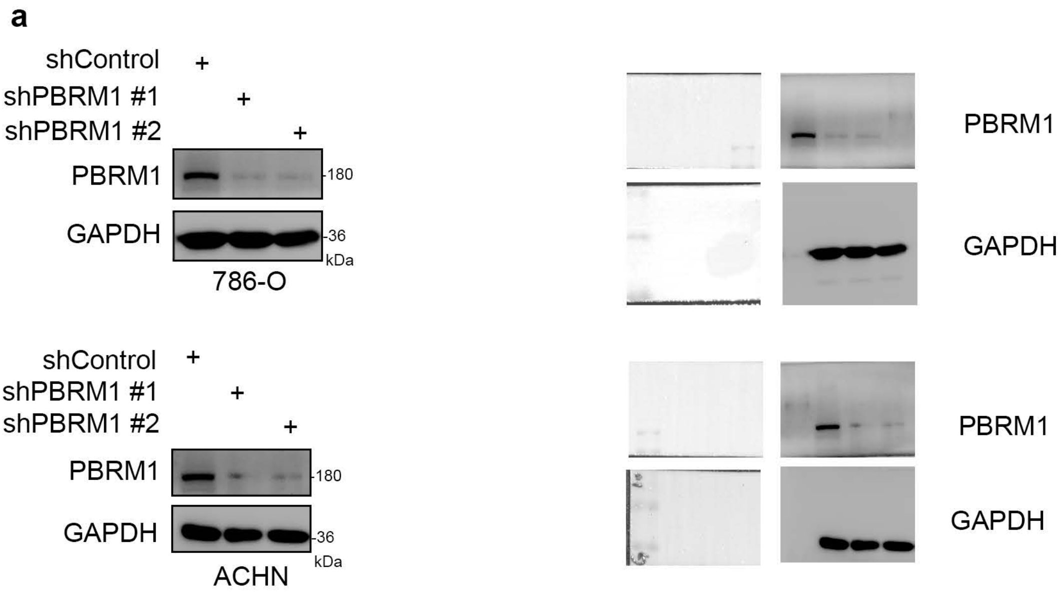

Supplementary figure 5

c

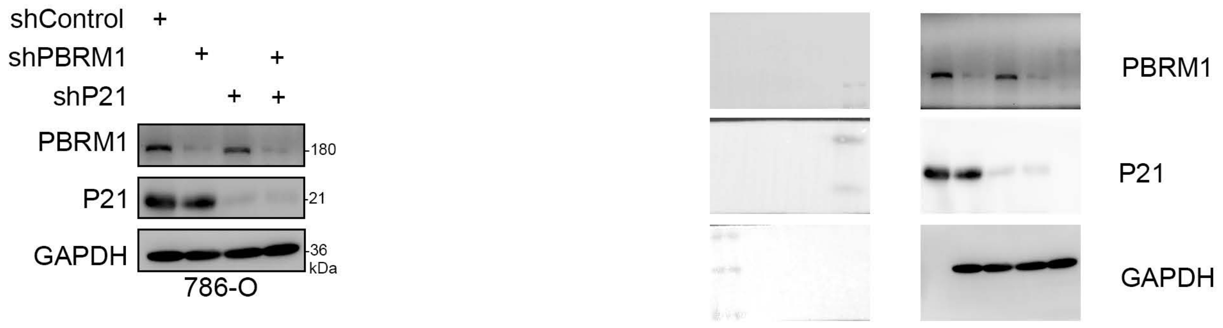

e

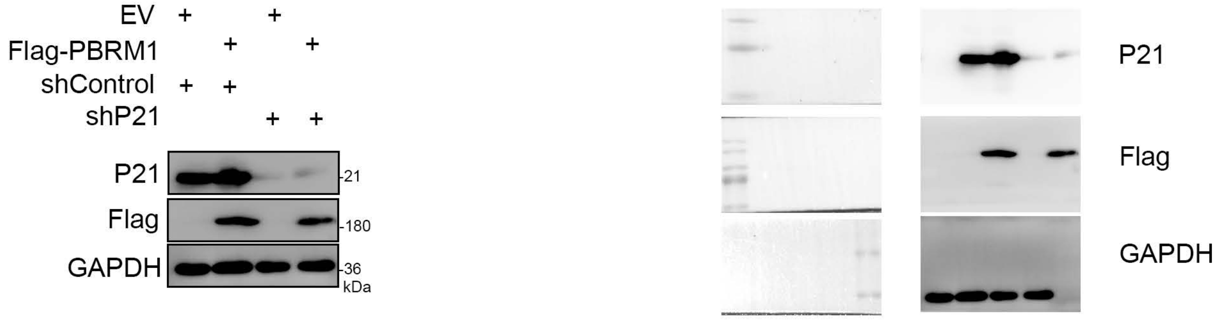

g

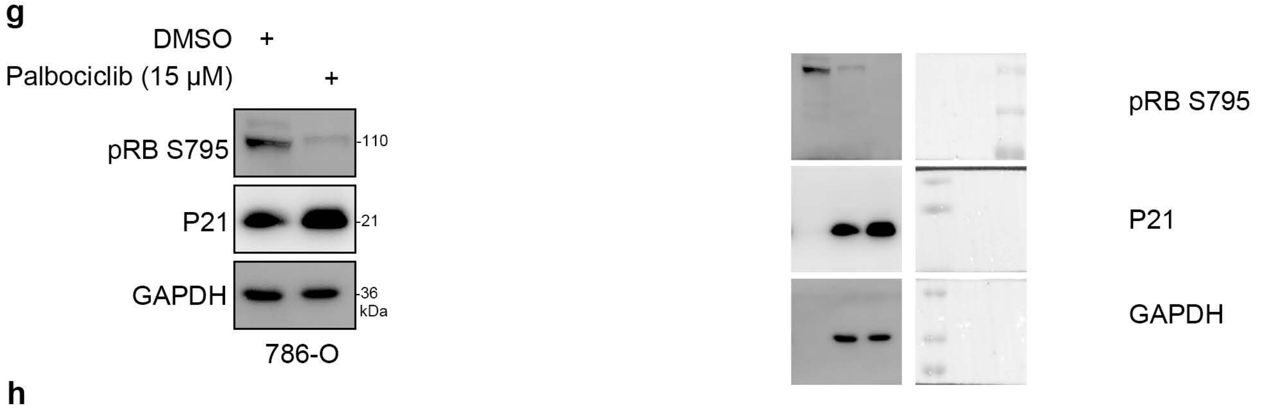

h

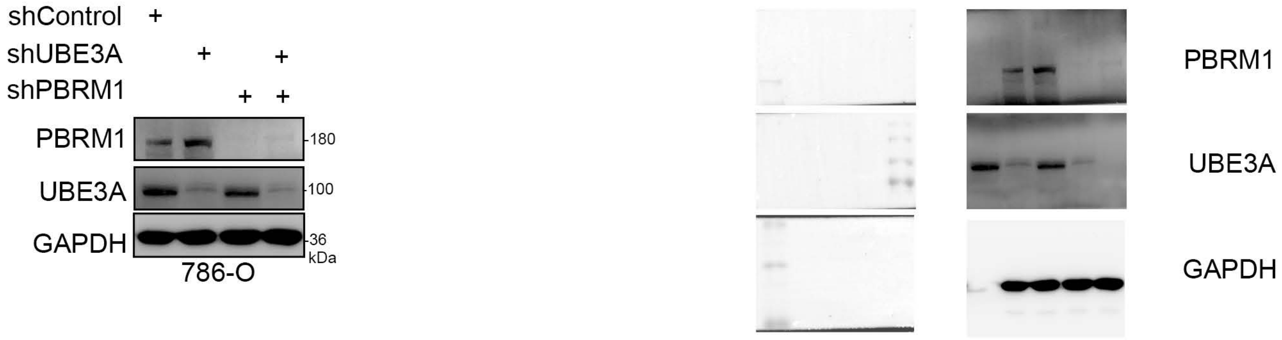

j

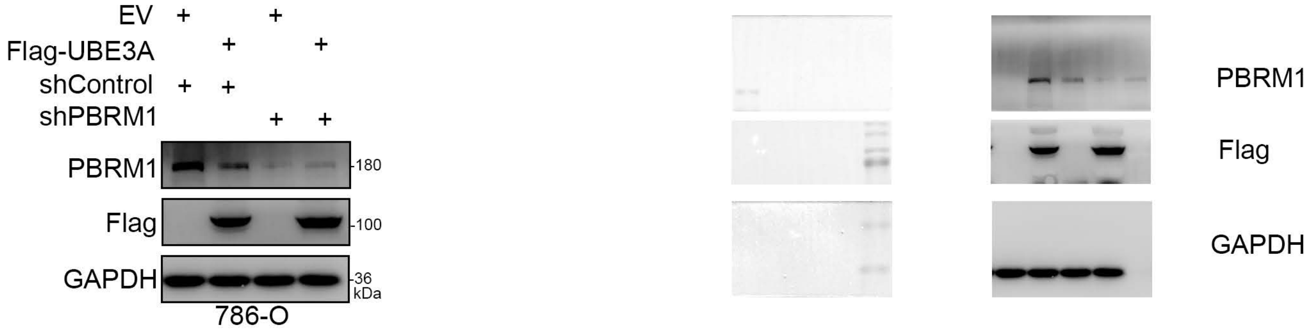

Supplement: Supplementary file 5 — Original western blot [file 41419_2022_4760_MOESM5_ESM.pdf]
