# Supplementary material for: The RBPJ/DAPK3/UBE3A signaling axis induces PBRM1 degradation to modulate the sensitivity of renal cell carcinoma to CDK4/6 inhibitors
Source: Cell Death Dis. 2022 Apr 2;13(4):295. doi: 10.1038/s41419-022-04760-6 (PMC8976838; doi:10.1038/s41419-022-04760-6)
Supplement: Supplementary file 6 — agreement from all author [file 41419_2022_4760_MOESM6_ESM.pdf]

## Re: Decision letter for CDDIS-21-2401RR: Accepted in Principle (change of authorship)

1封邮件

---

<xyeyylwt@csu.edu.cn> 今天 上午9:04

收件人： jinxinxy2 <jinxinxy2@csu.edu.cn>

I agree.

发自我的iPhone

------------------ Original ------------------

**From:** jinxinxy2@csu.edu.cn <jinxinxy2@csu.edu.cn>
**Date:** Fri,Mar 11,2022 7:44 AM
**To:** xyeyylwt@csu.edu.cn <xyeyylwt@csu.edu.cn>
**Subject:** Re: Decision letter for CDDIS-21-2401RR: Accepted in Principle (change of authorship)

Dr. Wentao Liu

The manuscript entitiled "The RBPJ/DAPK3/UBE3A signaling axis induces PBRM1 degradation to modulate the sensitivity of renal cell carcinoma to CDK4/6 inhibitors" has been submited to *Cell Death & Disease*. The original antuor list is "Wentao Liu, Bin Zhang, Dan Zhang, Feng Guo, Kun Ye, Liang Zhu, and Xin Jin". Dr.Dan Zhang performed a lot of experimental works in the revision stage of the article. Bases on her contribution to the article, we decided to add her to the article. The new author list is :Wentao Liu, Bin Zhang, Dan Zhang, Feng Guo, Kun Ye, Liang Zhu, and Xin Jin. If you agree/disaggree the change, please response me you decision.

Thanks
Xin Jin

-------- 原始邮件 --------
发件人： cddisease@springernature.com
日期： 2022年3月3日周四 傍晚5:11
收件人： jinxinxy2@csu.edu.cn
主 题： Decision letter for CDDIS-21-2401RR: Accepted in Principle
Manuscript Number: CDDIS-21-2401RR
Title: The RBPJ/DAPK3/UBE3A signaling axis induces PBRM1 degradation to modulate the sensitivity of renal cell carcinoma to CDK4/6 inhibitors
Authors: Wentao Liu, Bin Zhang, Dan Zhang, Feng Guo, Kun Ye, Liang Zhu, and Xin Jin

Dear Dr Jin,

I am very pleased to inform you that your manuscript CDDIS-21-2401RR entitled "The RBPJ/DAPK3/UBE3A signaling axis induces PBRM1 degradation to modulate the sensitivity of renal cell carcinoma to CDK4/6 inhibitors" has been **provisionally accepted** for publication in *Cell Death & Disease*.

**PLEASE READ THE FOLLOWING CAREFULLY**

Please note that we won't be able to officially confirm acceptance of your manuscript and proceed with the publication process until the final version of your manuscript has been submitted as per the instruction below.

*Cell Death & Disease* is a fully open access journal. Articles are made freely accessible on publication under a http://creativecommons.org/licenses/by/4.0"" target=""_blank""> CC BY license (Creative Commons Attribution 4.0 International License). This license allows maximum dissemination and re-use of open access materials and is preferred by many research funding bodies. For further information about article processing charges, open access funding, and advice and support from Nature Research, please visit https://www.nature.com/cddis/open-access

At acceptance, you will be provided with instructions for completing this CC BY license on behalf of all authors. This grants us the necessary permissions to publish your paper. Additionally, you will be asked to declare that all required third party permissions have been obtained, and to provide billing information in order to pay the article-processing charge (APC).

For more detailed information please check the https://www.nature.com/documents/cddis-gta.pdf"> Guide to the Authors.

**1. SUBMITTING YOUR FINAL FILES:**

Please submit your final manuscript here:
https://mts-cddis.nature.com/cgi-bin/main.plex?el=A6CA1Unx2C7BtFU7I3A9ftdain5ppQ62KFzO9JNgRP0wZ

- Files must not contain highlighting or tracked changes.
- Cell Death & Disease references should follow the Vancouver format. All authors should be listed for papers with up to six authors; for papers with more than six authors, the first six only should be listed, followed by et al.
- Figure and table legends must be included at the end of article file, after the references.
- Figure files must be uploaded as individual files and then included in the merged file, ensuring that figures with multiple panels are uploaded as one file (e.g. Fig. 1A, 1B uploaded as Fig. 1).
- Tables must be editable and either uploaded as separate files or included in the article file, after the references.
- All supplementary information, including supplementary figure and table legends, must be uploaded separately and not included in the article file.
- An Author Contribution Statement must be provided.
- All authors identified as 'corresponding author' should create and link their Open Researcher and Contributor Identifier (ORCID) with their account on the Manuscript Tracking System (MTS) prior to acceptance.

Please note that you must retain a copy of all uncropped **original experimental data at high resolution for five years, to be shown upon request by the journal. Failure to produce such data on request can result in a retraction by the journal.**

**- AUTHOR LIST:**
**Please review the author list to verify that it is complete and accurate. We ask that you consult with your co-authors to ensure that all names, affiliations, and titles are represented correctly. Should such details need correcting after publication, this will require a formal correction to be issued which should be avoided.**
**Please note, changes to authorship (addition or removal) will not be allowed once the manuscript has been formally accepted for publication.**

**For papers containing one or more consortia, all members of the consortium who contributed to the paper must be listed in the paper (i.e., print/online PDF). If necessary, individual authors can be listed in both the main author list and as a member of a consortium listed at the end of the paper. When submitting your revised manuscript via the online submission system, the consortium name should be entered as an author, together with the contact details of a nominated consortium representative. See our authorship policy and further consortia formatting guidelines, which should be adhered to prior to acceptance.**

**2. PUBLICATION REQUIREMENTS:**

**Pending submission of your final files, you will be contacted following the acceptance of your manuscript to complete the relevant publication and payment forms for your manuscript, hence you do not need to return any such forms with your final files.**

**3. ORCID:**

**Cell Death & Disease is committed to improving transparency in authorship. As part of our efforts in this direction, we are now requesting that all authors identified as 'corresponding author' create and link their Open Researcher and Contributor Identifier (ORCID) with their account on the Manuscript Tracking System (MTS) prior to acceptance. ORCID helps the scientific community achieve unambiguous attribution of all scholarly contributions. For more information please visit http://www.springernature.com/orcid**

**For all corresponding authors listed on the manuscript, please follow the instructions in the link below to link your ORCID to your account on our MTS before submitting the final version of the manuscript. If you do not yet have an ORCID you will be able to create one in minutes. https://www.springernature.com/gp/researchers/orcid/orcid-for-nature-research.**

**IMPORTANT: All authors identified as 'corresponding author' on the manuscript must follow these instructions. Non-corresponding authors do not have to link their ORCIDs but are encouraged to do so. Please note that it will not be possible to add/modify ORCIDs at proof. Thus, if they wish to have their ORCID added to the paper they must also follow the above procedure prior to acceptance.**

**To support ORCID's aims, we only allow a single ORCID identifier to be attached to one account. If you have any issues attaching an ORCID identifier to your MTS account, please contact the Platform Support Helpdesk.**

**You can now use a single sign-on for all your accounts, view the status of all your manuscript submissions and reviews, access usage statistics for your published articles and download a record of your refereeing activity for the Nature journals.**

**Sincerely,**

**Ygal Haupt**
**Receiving Editor**
**Cell Death & Disease**

--------------------------------------------------------------------------------

**Reviewer Comments:**

**This email has been sent through the NPG Manuscript Tracking System NY-610A-NPG&MTS**

***Confidentiality Statement:***

***This e-mail is confidential and subject to copyright. Any unauthorised use or disclosure of its contents is prohibited. If you have received this email in error please notify our Manuscript Tracking System Helpdesk team at http://platformsupport.nature.com .***

***Details of the confidentiality and pre-publicity policy may be found here http://www.nature.com/authors/policies/confidentiality.html***

**Privacy Policy | Update Profile**

# Re: Decision letter for CDDIS-21-2401RR: Accepted in Principle (change of authorship)

1封邮件

**张彬** <bluebin1020@qq.com> 今天 早上8:13

收件人： jinxinxy2 <jinxinxy2@csu.edu.cn>

I agree the change.

------------------ Original ------------------

**From:** jinxinxy2@csu.edu.cn <jinxinxy2@csu.edu.cn>
**Date:** Fri,Mar 11,2022 7:45 AM
**To:** 张彬 <bluebin1020@qq.com>
**Subject:** Re: Decision letter for CDDIS-21-2401RR: Accepted in Principle (change of authorship)

Dr. Bin Zhang

The manuscript entitiled "The RBPJ/DAPK3/UBE3A signaling axis induces PBRM1 degradation to modulate the sensitivity of renal cell carcinoma to CDK4/6 inhibitors" has been submited to *Cell Death & Disease.* The original antuor list is "Wentao Liu, Bin Zhang, Dan Zhang, Feng Guo, Kun Ye, Liang Zhu, and Xin Jin". Dr.Dan Zhang performed a lot of experimental works in the revision stage of the article. Bases on her contribution to the article, we decided to add her to the article. The new author list is :Wentao Liu, Bin Zhang, Dan Zhang, Feng Guo, Kun Ye, Liang Zhu, and Xin Jin. If you agree/disaggree the change, please response me you decision.

Thanks
Xin Jin

-------- 原始邮件 --------
发件人： cddisease@springernature.com
日期： 2022年3月3日周四 傍晚5:11
收件人： jinxinxy2@csu.edu.cn
主 题： Decision letter for CDDIS-21-2401RR: Accepted in Principle
Manuscript Number: CDDIS-21-2401RR
Title: The RBPJ/DAPK3/UBE3A signaling axis induces PBRM1 degradation to modulate the sensitivity of renal cell carcinoma to CDK4/6 inhibitors
Authors: Wentao Liu, Bin Zhang, Dan Zhang, Feng Guo, Kun Ye, Liang Zhu, and Xin Jin

Dear Dr Jin,

I am very pleased to inform you that your manuscript CDDIS-21-2401RR entitled "The RBPJ/DAPK3/UBE3A signaling axis induces PBRM1 degradation to modulate the sensitivity of renal cell carcinoma to CDK4/6 inhibitors" has been **provisionally accepted** for publication in *Cell Death & Disease*.

**PLEASE READ THE FOLLOWING CAREFULLY**

Please note that we won't be able to officially confirm acceptance of your manuscript and proceed with the publication process until the final version of your manuscript has been submitted as per the instruction below.

*Cell Death & Disease* is a fully open access journal. Articles are made freely accessible on publication under a http://creativecommons.org/licenses/by/4.0"" target=""_blank""> CC BY license (Creative Commons Attribution 4.0 International License). This license allows maximum dissemination and re-use of open access materials and is preferred by many research funding bodies. For further information about article processing charges, open access funding, and advice and support from Nature Research, please visit https://www.nature.com/cddis/open-access

At acceptance, you will be provided with instructions for completing this CC BY license on behalf of all authors. This grants us the necessary permissions to publish your paper. Additionally, you will be asked to declare that all required third party permissions have been obtained, and to provide billing information in order to pay the article-processing charge (APC).

For more detailed information please check the https://www.nature.com/documents/cddis-gta.pdf"> Guide to the Authors.

**1. SUBMITTING YOUR FINAL FILES:**

Please submit your final manuscript here:
https://mts-cddis.nature.com/cgi-bin/main.plex?el=A6CA1Unx2C7BtFU7I3A9ftdain5ppQ62KFzO9JNgRP0wZ

- Files must not contain highlighting or tracked changes.
- Cell Death & Disease references should follow the Vancouver format. All authors should be listed for papers with up to six authors; for papers with more than six authors, the first six only should be listed, followed by et al.
- Figure and table legends must be included at the end of article file, after the references.
- Figure files must be uploaded as individual files and then included in the merged file, ensuring that figures with multiple panels are uploaded as one file (e.g. Fig. 1A, 1B uploaded as Fig. 1).
- Tables must be editable and either uploaded as separate files or included in the article file, after the references.
- All supplementary information, including supplementary figure and table legends, must be uploaded separately and not included in the article file.
- An Author Contribution Statement must be provided.
- All authors identified as 'corresponding author' should create and link their Open Researcher and Contributor Identifier (ORCID) with their account on the Manuscript Tracking System (MTS) prior to acceptance.

Please note that you must retain a copy of all uncropped **original experimental data at high resolution for five years, to be shown upon request by the journal. Failure to produce such data on request can result in a retraction by the journal.**

**- AUTHOR LIST:**
**Please review the author list to verify that it is complete and accurate. We ask that you consult with your co-authors to ensure that all names, affiliations, and titles are represented correctly. Should such details need correcting after publication, this will require a formal correction to be issued which should be avoided.**
**Please note, changes to authorship (addition or removal) will not be allowed once the manuscript has been formally accepted for publication.**

**For papers containing one or more consortia, all members of the consortium who contributed to the paper must be listed in the paper (i.e., print/online PDF). If necessary, individual authors can be listed in both the main author list and as a member of a consortium listed at the end of the paper. When submitting your revised manuscript via the online submission system, the consortium name should be entered as an author, together with the contact details of a nominated consortium representative. See our authorship policy and further consortia formatting guidelines, which should be adhered to prior to acceptance.**

**2. PUBLICATION REQUIREMENTS:**

**Pending submission of your final files, you will be contacted following the acceptance of your manuscript to complete the relevant publication and payment forms for your manuscript, hence you do not need to return any such forms with your final files.**

**3. ORCID:**

**Cell Death & Disease is committed to improving transparency in authorship. As part of our efforts in this direction, we are now requesting that all authors identified as 'corresponding author' create and link their Open Researcher and Contributor Identifier (ORCID) with their account on the Manuscript Tracking System (MTS) prior to acceptance. ORCID helps the scientific community achieve unambiguous attribution of all scholarly contributions. For more information please visit http://www.springernature.com/orcid**

**For all corresponding authors listed on the manuscript, please follow the instructions in the link below to link your ORCID to your account on our MTS before submitting the final version of the manuscript. If you do not yet have an ORCID you will be able to create one in minutes. https://www.springernature.com/gp/researchers/orcid/orcid-for-nature-research.**

**IMPORTANT: All authors identified as 'corresponding author' on the manuscript must follow these instructions. Non-corresponding authors do not have to link their ORCIDs but are encouraged to do so. Please note that it will not be possible to add/modify ORCIDs at proof. Thus, if they wish to have their ORCID added to the paper they must also follow the above procedure prior to acceptance.**

**To support ORCID's aims, we only allow a single ORCID identifier to be attached to one account. If**

you have any issues attaching an ORCID identifier to your MTS account, please contact the Platform Support Helpdesk.

You can now use a single sign-on for all your accounts, view the status of all your manuscript submissions and reviews, access usage statistics for your published articles and download a record of your refereeing activity for the Nature journals.

Sincerely,

Ygal Haupt
Receiving Editor
Cell Death & Disease

--------------------------------------------------------------------------------

Reviewer Comments:

This email has been sent through the NPG Manuscript Tracking System NY-610A-NPG&MTS

*Confidentiality Statement:*

*This e-mail is confidential and subject to copyright. Any unauthorised use or disclosure of its contents is prohibited. If you have received this email in error please notify our Manuscript Tracking System Helpdesk team at http://platformsupport.nature.com .*

*Details of the confidentiality and pre-publicity policy may be found here http://www.nature.com/authors/policies/confidentiality.html*

Privacy Policy | Update Profile

# Re: Decision letter for CDDIS-21-2401RR: Accepted in Principle (change of authorship)

1封邮件

**reseda_zd** <369331213@qq.com> 今天 上午10:10

收件人： jinxinxy2@csu.edu.cn <jinxinxy2@csu.edu.cn>

Professor Jin,
Thank you for the information, and I agree with the desicion.
Thanks,
Dan Zhang.

---Original---

**From:** "jinxinxy2@csu.edu.cn"<jinxinxy2@csu.edu.cn>
**Date:** Fri, Mar 11, 2022 07:46 AM
**To:** "reseda_zd"<369331213@qq.com>;
**Subject:** Decision letter for CDDIS-21-2401RR: Accepted in Principle (change of authorship)

Dr. Dan Zhang

The manuscript entitiled "The RBPJ/DAPK3/UBE3A signaling axis induces PBRM1 degradation to modulate the sensitivity of renal cell carcinoma to CDK4/6 inhibitors" has been submited to *Cell Death & Disease*. The original antuor list is "Wentao Liu, Bin Zhang, Dan Zhang, Feng Guo, Kun Ye, Liang Zhu, and Xin Jin". Dr.Dan Zhang performed a lot of experimental works in the revision stage of the article. Bases on her contribution to the article, we decided to add her to the article. The new author list is :Wentao Liu, Bin Zhang, Dan Zhang, Feng Guo, Kun Ye, Liang Zhu, and Xin Jin. If you agree/disaggree the change, please response me you decision.

Thanks
Xin Jin

-------- 原始邮件 --------
发件人： cddisease@springernature.com
日期： 2022年3月3日周四 傍晚5:11
收件人： jinxinxy2@csu.edu.cn
主 题： Decision letter for CDDIS-21-2401RR: Accepted in Principle
Manuscript Number: CDDIS-21-2401RR
Title: The RBPJ/DAPK3/UBE3A signaling axis induces PBRM1 degradation to modulate the sensitivity of renal cell carcinoma to CDK4/6 inhibitors
Authors: Wentao Liu, Bin Zhang, Dan Zhang, Feng Guo, Kun Ye, Liang Zhu, and Xin Jin

Dear Dr Jin,

I am very pleased to inform you that your manuscript CDDIS-21-2401RR entitled "The RBPJ/DAPK3/UBE3A signaling axis induces PBRM1 degradation to modulate the sensitivity of renal cell carcinoma to CDK4/6 inhibitors" has been **provisionally accepted** for publication in *Cell Death & Disease*.

**PLEASE READ THE FOLLOWING CAREFULLY**

Please note that we won’t be able to officially confirm acceptance of your manuscript and proceed with the publication process until the final version of your manuscript has been submitted as per the instruction below.

*Cell Death & Disease* is a fully open access journal. Articles are made freely accessible on publication under a http://creativecommons.org/licenses/by/4.0"" target=""_blank""> CC BY license (Creative Commons Attribution 4.0 International License). This license allows maximum dissemination and re-use of open access materials and is preferred by many research funding bodies. For further information about article processing charges, open access funding, and advice and support from Nature Research, please visit https://www.nature.com/cddis/open-access

At acceptance, you will be provided with instructions for completing this CC BY license on behalf of all authors. This grants us the necessary permissions to publish your paper. Additionally, you will be asked to declare that all required third party permissions have been obtained, and to provide billing information in order to pay the article-processing charge (APC).

For more detailed information please check the https://www.nature.com/documents/cddis-gta.pdf"> Guide to the Authors.

**1. SUBMITTING YOUR FINAL FILES:**

Please submit your final manuscript here:
https://mts-cddis.nature.com/cgi-bin/main.plex?el=A6CA1Unx2C7BtFU7I3A9ftdain5ppQ62KFzO9JNgRP0wZ

- Files must not contain highlighting or tracked changes.
- Cell Death & Disease references should follow the Vancouver format. All authors should be listed for papers with up to six authors; for papers with more than six authors, the first six only should be listed, followed by et al.
- Figure and table legends must be included at the end of article file, after the references.
- Figure files must be uploaded as individual files and then included in the merged file, ensuring that figures with multiple panels are uploaded as one file (e.g. Fig. 1A, 1B uploaded as Fig. 1).
- Tables must be editable and either uploaded as separate files or included in the article file, after the references.
- All supplementary information, including supplementary figure and table legends, must be uploaded separately and not included in the article file.
- An Author Contribution Statement must be provided.
- All authors identified as ‘corresponding author’ should create and link their Open Researcher and Contributor Identifier (ORCID) with their account on the Manuscript Tracking System (MTS) prior to acceptance.

Please note that you must retain a copy of all uncropped **original experimental data at high resolution for five years, to be shown upon request by the journal. Failure to produce such data on request can result in a retraction by the journal.**

**- AUTHOR LIST:**
**Please review the author list to verify that it is complete and accurate. We ask that you consult with your co-authors to ensure that all names, affiliations, and titles are represented correctly. Should such details need correcting after publication, this will require a formal correction to be issued which should be avoided.**
**Please note, changes to authorship (addition or removal) will not be allowed once the manuscript has been formally accepted for publication.**

**For papers containing one or more consortia, all members of the consortium who contributed to the paper must be listed in the paper (i.e., print/online PDF). If necessary, individual authors can be listed in both the main author list and as a member of a consortium listed at the end of the paper. When submitting your revised manuscript via the online submission system, the consortium name should be entered as an author, together with the contact details of a nominated consortium representative. See our authorship policy and further consortia formatting guidelines, which should be adhered to prior to acceptance.**

**2. PUBLICATION REQUIREMENTS:**

**Pending submission of your final files, you will be contacted following the acceptance of your manuscript to complete the relevant publication and payment forms for your manuscript, hence you do not need to return any such forms with your final files.**

**3. ORCID:**

**Cell Death & Disease is committed to improving transparency in authorship. As part of our efforts in this direction, we are now requesting that all authors identified as 'corresponding author' create and link their Open Researcher and Contributor Identifier (ORCID) with their account on the Manuscript Tracking System (MTS) prior to acceptance. ORCID helps the scientific community achieve unambiguous attribution of all scholarly contributions. For more information please visit http://www.springernature.com/orcid**

**For all corresponding authors listed on the manuscript, please follow the instructions in the link below to link your ORCID to your account on our MTS before submitting the final version of the manuscript. If you do not yet have an ORCID you will be able to create one in minutes. https://www.springernature.com/gp/researchers/orcid/orcid-for-nature-research.**

**IMPORTANT: All authors identified as 'corresponding author' on the manuscript must follow these instructions. Non-corresponding authors do not have to link their ORCIDs but are encouraged to do so. Please note that it will not be possible to add/modify ORCIDs at proof. Thus, if they wish to have their ORCID added to the paper they must also follow the above procedure prior to acceptance.**

**To support ORCID's aims, we only allow a single ORCID identifier to be attached to one account. If**

you have any issues attaching an ORCID identifier to your MTS account, please contact the Platform Support Helpdesk.

You can now use a single sign-on for all your accounts, view the status of all your manuscript submissions and reviews, access usage statistics for your published articles and download a record of your refereeing activity for the Nature journals.

Sincerely,

Ygal Haupt
Receiving Editor
Cell Death & Disease

---------------------------------------------------------------------------------

Reviewer Comments:

This email has been sent through the NPG Manuscript Tracking System NY-610A-NPG&MTS

*Confidentiality Statement:*

*This e-mail is confidential and subject to copyright. Any unauthorised use or disclosure of its contents is prohibited. If you have received this email in error please notify our Manuscript Tracking System Helpdesk team at http://platformsupport.nature.com .*

*Details of the confidentiality and pre-publicity policy may be found here http://www.nature.com/authors/policies/confidentiality.html*

Privacy Policy | Update Profile

## Re:Decision letter for CDDIS-21-2401RR: Accepted in Principle (change of authorship)

1封邮件

**郭丰** <guofengunion@hust.edu.cn> 今天 中午11:00

收件人： jinxinxy2@csu.edu.cn <jinxinxy2@csu.edu.cn>

I totally agree with adjusting the order of authors, thanks

---

发件人："jinxinxy2@csu.edu.cn" <jinxinxy2@csu.edu.cn>
发送日期：2022-03-11 07:46:41
收件人："guofengunion@hust.edu.cn" <guofengunion@hust.edu.cn>
抄送人：
主 题：Decision letter for CDDIS-21-2401RR: Accepted in Principle (change of authorship)

Dr. Feng Guo

The manuscript entitiled "The RBPJ/DAPK3/UBE3A signaling axis induces PBRM1 degradation to modulate the sensitivity of renal cell carcinoma to CDK4/6 inhibitors" has been submited to *Cell Death & Disease*. The original antuor list is "Wentao Liu, Bin Zhang, Dan Zhang, Feng Guo, Kun Ye, Liang Zhu, and Xin Jin". Dr.Dan Zhang performed a lot of experimental works in the revision stage of the article. Bases on her contribution to the article, we decided to add her to the article. The new author list is :Wentao Liu, Bin Zhang, Dan Zhang, Feng Guo, Kun Ye, Liang Zhu, and Xin Jin. If you agree/disaggree the change, please response me you decision.

Thanks
Xin Jin

-------- 原始邮件 --------
发件人： cddisease@springernature.com
日期： 2022年3月3日周四 傍晚5:11
收件人： jinxinxy2@csu.edu.cn
主 题： Decision letter for CDDIS-21-2401RR: Accepted in Principle
Manuscript Number: CDDIS-21-2401RR
Title: The RBPJ/DAPK3/UBE3A signaling axis induces PBRM1 degradation to modulate the sensitivity of renal cell carcinoma to CDK4/6 inhibitors
Authors: Wentao Liu, Bin Zhang, Dan Zhang, Feng Guo, Kun Ye, Liang Zhu, and Xin Jin

Dear Dr Jin,

I am very pleased to inform you that your manuscript CDDIS-21-2401RR entitled "The

RBPJ/DAPK3/UBE3A signaling axis induces PBRM1 degradation to modulate the sensitivity of renal cell carcinoma to CDK4/6 inhibitors" has been **provisionally accepted** for publication in *Cell Death & Disease*.

**PLEASE READ THE FOLLOWING CAREFULLY**

Please note that we won't be able to officially confirm acceptance of your manuscript and proceed with the publication process until the final version of your manuscript has been submitted as per the instruction below.

*Cell Death & Disease* is a fully open access journal. Articles are made freely accessible on publication under a http://creativecommons.org/licenses/by/4.0"" target=""_blank""> CC BY license (Creative Commons Attribution 4.0 International License). This license allows maximum dissemination and re-use of open access materials and is preferred by many research funding bodies. For further information about article processing charges, open access funding, and advice and support from Nature Research, please visit https://www.nature.com/cddis/open-access

At acceptance, you will be provided with instructions for completing this CC BY license on behalf of all authors. This grants us the necessary permissions to publish your paper. Additionally, you will be asked to declare that all required third party permissions have been obtained, and to provide billing information in order to pay the article-processing charge (APC).

For more detailed information please check the https://www.nature.com/documents/cddis-gta.pdf"> Guide to the Authors.

**1. SUBMITTING YOUR FINAL FILES:**

Please submit your final manuscript here:
https://mts-cddis.nature.com/cgi-bin/main.plex?el=A6CA1Unx2C7BtFU7I3A9ftdain5ppQ62KFzO9JNgRP0wZ

- Files must not contain highlighting or tracked changes.
- Cell Death & Disease references should follow the Vancouver format. All authors should be listed for papers with up to six authors; for papers with more than six authors, the first six only should be listed, followed by et al.
- Figure and table legends must be included at the end of article file, after the references.
- Figure files must be uploaded as individual files and then included in the merged file, ensuring that figures with multiple panels are uploaded as one file (e.g. Fig. 1A, 1B uploaded as Fig. 1).
- Tables must be editable and either uploaded as separate files or included in the article file, after the references.
- All supplementary information, including supplementary figure and table legends, must be uploaded separately and not included in the article file.
- An Author Contribution Statement must be provided.
- All authors identified as 'corresponding author' should create and link their Open Researcher and Contributor Identifier (ORCID) with their account on the Manuscript Tracking System (MTS) prior to acceptance.

Please note that you must retain a copy of all uncropped **original experimental data at high resolution**

for five years, to be shown upon request by the journal. Failure to produce such data on request can result in a retraction by the journal.

- AUTHOR LIST:
Please review the author list to verify that it is complete and accurate. We ask that you consult with your co-authors to ensure that all names, affiliations, and titles are represented correctly. Should such details need correcting after publication, this will require a formal correction to be issued which should be avoided.
Please note, changes to authorship (addition or removal) will not be allowed once the manuscript has been formally accepted for publication.

For papers containing one or more consortia, all members of the consortium who contributed to the paper must be listed in the paper (i.e., print/online PDF). If necessary, individual authors can be listed in both the main author list and as a member of a consortium listed at the end of the paper. When submitting your revised manuscript via the online submission system, the consortium name should be entered as an author, together with the contact details of a nominated consortium representative. See our authorship policy and further consortia formatting guidelines, which should be adhered to prior to acceptance.

2. PUBLICATION REQUIREMENTS:

Pending submission of your final files, you will be contacted following the acceptance of your manuscript to complete the relevant publication and payment forms for your manuscript, hence you do not need to return any such forms with your final files.

3. ORCID:

Cell Death & Disease is committed to improving transparency in authorship. As part of our efforts in this direction, we are now requesting that all authors identified as 'corresponding author' create and link their Open Researcher and Contributor Identifier (ORCID) with their account on the Manuscript Tracking System (MTS) prior to acceptance. ORCID helps the scientific community achieve unambiguous attribution of all scholarly contributions. For more information please visit http://www.springernature.com/orcid

For all corresponding authors listed on the manuscript, please follow the instructions in the link below to link your ORCID to your account on our MTS before submitting the final version of the manuscript. If you do not yet have an ORCID you will be able to create one in minutes.
https://www.springernature.com/gp/researchers/orcid/orcid-for-nature-research.

IMPORTANT: All authors identified as 'corresponding author' on the manuscript must follow these instructions. Non-corresponding authors do not have to link their ORCIDs but are encouraged to do so. Please note that it will not be possible to add/modify ORCIDs at proof. Thus, if they wish to have their ORCID added to the paper they must also follow the above procedure prior to acceptance.

To support ORCID's aims, we only allow a single ORCID identifier to be attached to one account. If you have any issues attaching an ORCID identifier to your MTS account, please contact the Platform Support Helpdesk.

You can now use a single sign-on for all your accounts, view the status of all your manuscript submissions and reviews, access usage statistics for your published articles and download a record of your refereeing activity for the Nature journals.

Sincerely,

Ygal Haupt
Receiving Editor
Cell Death & Disease

------------------------------------------------------------------------------

Reviewer Comments:

This email has been sent through the NPG Manuscript Tracking System NY-610A-NPG&MTS

*Confidentiality Statement:*

*This e-mail is confidential and subject to copyright. Any unauthorised use or disclosure of its contents is prohibited. If you have received this email in error please notify our Manuscript Tracking System Helpdesk team at http://platformsupport.nature.com .*

*Details of the confidentiality and pre-publicity policy may be found here http://www.nature.com/authors/policies/confidentiality.html*

Privacy Policy | Update Profile

# Re: Decision letter for CDDIS-21-2401RR: Accepted in Principle (change of authorship)

1封邮件

**叶昆** <yk1198703592@qq.com> 今天 上午10:03

收件人： jinxinxy2@csu.edu.cn <jinxinxy2@csu.edu.cn>

Agree

---Original---

**From:** "jinxinxy2@csu.edu.cn"<jinxinxy2@csu.edu.cn>
**Date:** Fri, Mar 11, 2022 07:47 AM
**To:** "叶昆"<yk1198703592@qq.com>;
**Subject:** Decision letter for CDDIS-21-2401RR: Accepted in Principle (change of authorship)

Dr. Kun YE

The manuscript entitiled  "The RBPJ/DAPK3/UBE3A signaling axis induces PBRM1 degradation to modulate the sensitivity of renal cell carcinoma to CDK4/6 inhibitors" has been submited to *Cell Death & Disease*. The original antuor list is "Wentao Liu, Bin Zhang, Dan Zhang, Feng Guo, Kun Ye, Liang Zhu, and Xin Jin". Dr.Dan Zhang performed a lot of experimental works in the revision stage of the article. Bases on her contribution to the article, we decided to add her to the article. The new author list is :Wentao Liu, Bin Zhang, Dan Zhang, Feng Guo, Kun Ye, Liang Zhu, and Xin Jin. If you agree/disaggree the change, please response me you decision.

Thanks
Xin Jin

-------- 原始邮件 --------
发件人： cddisease@springernature.com
日期： 2022年3月3日周四 傍晚5:11
收件人： jinxinxy2@csu.edu.cn
主 题： Decision letter for CDDIS-21-2401RR: Accepted in Principle
Manuscript Number: CDDIS-21-2401RR
Title: The RBPJ/DAPK3/UBE3A signaling axis induces PBRM1 degradation to modulate the sensitivity of renal cell carcinoma to CDK4/6 inhibitors
Authors: Wentao Liu, Bin Zhang, Dan Zhang, Feng Guo, Kun Ye, Liang Zhu, and Xin Jin

Dear Dr Jin,

I am very pleased to inform you that your manuscript CDDIS-21-2401RR entitled "The

RBPJ/DAPK3/UBE3A signaling axis induces PBRM1 degradation to modulate the sensitivity of renal cell carcinoma to CDK4/6 inhibitors" has been **provisionally accepted** for publication in *Cell Death & Disease*.

**PLEASE READ THE FOLLOWING CAREFULLY**

Please note that we won't be able to officially confirm acceptance of your manuscript and proceed with the publication process until the final version of your manuscript has been submitted as per the instruction below.

*Cell Death & Disease* is a fully open access journal. Articles are made freely accessible on publication under a http://creativecommons.org/licenses/by/4.0"" target=""_blank""> CC BY license (Creative Commons Attribution 4.0 International License). This license allows maximum dissemination and re-use of open access materials and is preferred by many research funding bodies. For further information about article processing charges, open access funding, and advice and support from Nature Research, please visit https://www.nature.com/cddis/open-access

At acceptance, you will be provided with instructions for completing this CC BY license on behalf of all authors. This grants us the necessary permissions to publish your paper. Additionally, you will be asked to declare that all required third party permissions have been obtained, and to provide billing information in order to pay the article-processing charge (APC).

For more detailed information please check the https://www.nature.com/documents/cddis-gta.pdf"> Guide to the Authors.

**1. SUBMITTING YOUR FINAL FILES:**

Please submit your final manuscript here:
https://mts-cddis.nature.com/cgi-bin/main.plex?el=A6CA1Unx2C7BtFU7I3A9ftdain5ppQ62KFzO9JNgRP0wZ

- Files must not contain highlighting or tracked changes.
- Cell Death & Disease references should follow the Vancouver format. All authors should be listed for papers with up to six authors; for papers with more than six authors, the first six only should be listed, followed by et al.
- Figure and table legends must be included at the end of article file, after the references.
- Figure files must be uploaded as individual files and then included in the merged file, ensuring that figures with multiple panels are uploaded as one file (e.g. Fig. 1A, 1B uploaded as Fig. 1).
- Tables must be editable and either uploaded as separate files or included in the article file, after the references.
- All supplementary information, including supplementary figure and table legends, must be uploaded separately and not included in the article file.
- An Author Contribution Statement must be provided.
- All authors identified as 'corresponding author' should create and link their Open Researcher and Contributor Identifier (ORCID) with their account on the Manuscript Tracking System (MTS) prior to acceptance.

Please note that you must retain a copy of all uncropped **original experimental data at high resolution**

for five years, to be shown upon request by the journal. Failure to produce such data on request can result in a retraction by the journal.

- AUTHOR LIST:
Please review the author list to verify that it is complete and accurate. We ask that you consult with your co-authors to ensure that all names, affiliations, and titles are represented correctly. Should such details need correcting after publication, this will require a formal correction to be issued which should be avoided.
Please note, changes to authorship (addition or removal) will not be allowed once the manuscript has been formally accepted for publication.

For papers containing one or more consortia, all members of the consortium who contributed to the paper must be listed in the paper (i.e., print/online PDF). If necessary, individual authors can be listed in both the main author list and as a member of a consortium listed at the end of the paper. When submitting your revised manuscript via the online submission system, the consortium name should be entered as an author, together with the contact details of a nominated consortium representative. See our authorship policy and further consortia formatting guidelines, which should be adhered to prior to acceptance.

2. PUBLICATION REQUIREMENTS:

Pending submission of your final files, you will be contacted following the acceptance of your manuscript to complete the relevant publication and payment forms for your manuscript, hence you do not need to return any such forms with your final files.

3. ORCID:

Cell Death & Disease is committed to improving transparency in authorship. As part of our efforts in this direction, we are now requesting that all authors identified as 'corresponding author' create and link their Open Researcher and Contributor Identifier (ORCID) with their account on the Manuscript Tracking System (MTS) prior to acceptance. ORCID helps the scientific community achieve unambiguous attribution of all scholarly contributions. For more information please visit
http://www.springernature.com/orcid

For all corresponding authors listed on the manuscript, please follow the instructions in the link below to link your ORCID to your account on our MTS before submitting the final version of the manuscript. If you do not yet have an ORCID you will be able to create one in minutes.
https://www.springernature.com/gp/researchers/orcid/orcid-for-nature-research.

IMPORTANT: All authors identified as 'corresponding author' on the manuscript must follow these instructions. Non-corresponding authors do not have to link their ORCIDs but are encouraged to do so. Please note that it will not be possible to add/modify ORCIDs at proof. Thus, if they wish to have their ORCID added to the paper they must also follow the above procedure prior to acceptance.

To support ORCID's aims, we only allow a single ORCID identifier to be attached to one account. If you have any issues attaching an ORCID identifier to your MTS account, please contact the Platform Support Helpdesk.

**You can now use a single sign-on for all your accounts, view the status of all your manuscript submissions and reviews, access usage statistics for your published articles and download a record of your refereeing activity for the Nature journals.**

**Sincerely,**

**Ygal Haupt**
**Receiving Editor**
**Cell Death & Disease**

---------------------------------------------------------------------------------

**Reviewer Comments:**

**This email has been sent through the NPG Manuscript Tracking System NY-610A-NPG&MTS**

***Confidentiality Statement:***

***This e-mail is confidential and subject to copyright. Any unauthorised use or disclosure of its contents is prohibited. If you have received this email in error please notify our Manuscript Tracking System Helpdesk team at http://platformsupport.nature.com .***

***Details of the confidentiality and pre-publicity policy may be found here http://www.nature.com/authors/policies/confidentiality.html***

**Privacy Policy | Update Profile**

# Re: Decision letter for CDDIS-21-2401RR: Accepted in Principle (change of authorship)

1封邮件

**朱梁** <zhuliang@csu.edu.cn> 今天 早上8:58

收件人： jinxinxy2@csu.edu.cn <jinxinxy2@csu.edu.cn>

Yes, I agree this change.

Zhu

-----原始邮件-----

**发件人:**"jinxinxy2@csu.edu.cn" <jinxinxy2@csu.edu.cn>

**发送时间:**2022-03-11 07:48:15 (星期五)

**收件人:** "zhuliang@csu.edu.cn" <zhuliang@csu.edu.cn>

**抄送:**

**主题:** Decision letter for CDDIS-21-2401RR: Accepted in Principle (change of authorship)

Dr. Liang Zhu

The manuscript entitiled  "The RBPJ/DAPK3/UBE3A signaling axis induces PBRM1 degradation to modulate the sensitivity of renal cell carcinoma to CDK4/6 inhibitors" has been submited to *Cell Death & Disease*. The original antuor list is "Wentao Liu, Bin Zhang, Dan Zhang, Feng Guo, Kun Ye, Liang Zhu, and Xin Jin". Dr.Dan Zhang performed a lot of experimental works in the revision stage of the article. Bases on her contribution to the article, we decided to add her to the article. The new author list is :Wentao Liu, Bin Zhang, Dan Zhang, Feng Guo, Kun Ye, Liang Zhu, and Xin Jin. If you agree/disaggree the change, please response me you decision.

Thanks

Xin Jin

-------- 原始邮件 --------

发件人： cddisease@springernature.com

日期： 2022年3月3日周四 傍晚5:11

收件人： jinxinxy2@csu.edu.cn

主 题： Decision letter for CDDIS-21-2401RR: Accepted in Principle

Manuscript Number: CDDIS-21-2401RR

Title: The RBPJ/DAPK3/UBE3A signaling axis induces PBRM1 degradation to modulate the sensitivity of renal cell carcinoma to CDK4/6 inhibitors

Authors: Wentao Liu, Bin Zhang, Dan Zhang, Feng Guo, Kun Ye, Liang Zhu, and Xin Jin

Dear Dr Jin,

I am very pleased to inform you that your manuscript CDDIS-21-2401RR entitled "The RBPJ/DAPK3/UBE3A signaling axis induces PBRM1 degradation to modulate the sensitivity of renal cell carcinoma to CDK4/6 inhibitors" has been **provisionally accepted** for publication in *Cell Death & Disease*.

**PLEASE READ THE FOLLOWING CAREFULLY**

Please note that we won’t be able to officially confirm acceptance of your manuscript and proceed with the publication process until the final version of your manuscript has been submitted as per the instruction below.

*Cell Death & Disease* is a fully open access journal. Articles are made freely accessible on publication under a http://creativecommons.org/licenses/by/4.0"" target=""_blank""> CC BY license (Creative Commons Attribution 4.0 International License). This license allows maximum dissemination and re-use of open access materials and is preferred by many research funding bodies. For further information about article processing charges, open access funding, and advice and support from Nature Research, please visit https://www.nature.com/cddis/open-access

At acceptance, you will be provided with instructions for completing this CC BY license on behalf of all authors. This grants us the necessary permissions to publish your paper. Additionally, you will be asked to declare that all required third party permissions have been obtained, and to provide billing information in order to pay the article-processing charge (APC).

For more detailed information please check the https://www.nature.com/documents/cddis-gta.pdf" data-ke-src=">https://www.nature.com/documents/cddis-gta.pdf</a>"> Guide to the Authors.

**1. SUBMITTING YOUR FINAL FILES:**

Please submit your final manuscript here:
https://mts-cddis.nature.com/cgi-bin/main.plex?el=A6CA1Unx2C7BtFU7I3A9ftdain5ppQ62KFzO9JNgRP0wZ

- Files must not contain highlighting or tracked changes.
- Cell Death & Disease references should follow the Vancouver format. All authors should be listed for papers with up to six authors; for papers with more than six authors, the first six only should be listed, followed by et al.
- Figure and table legends must be included at the end of article file, after the references.
- Figure files must be uploaded as individual files and then included in the merged file, ensuring that figures with multiple panels are uploaded as one file (e.g. Fig. 1A, 1B uploaded as Fig. 1).
- Tables must be editable and either uploaded as separate files or included in the article file, after the references.
- All supplementary information, including supplementary figure and table legends, must be uploaded separately and not included in the article file.
- An Author Contribution Statement must be provided.
- All authors identified as ‘corresponding author’ should create and link their Open Researcher and Contributor Identifier (ORCID) with their account on the Manuscript Tracking System (MTS) prior to acceptance.

Please note that you must retain a copy of all uncropped **original experimental data at high resolution for five years, to be shown upon request by the journal. Failure to produce such data on request can result in a retraction by the journal.**

**- AUTHOR LIST:**
**Please review the author list to verify that it is complete and accurate. We ask that you consult with your co-authors to ensure that all names, affiliations, and titles are represented correctly. Should such details need correcting after publication, this will require a formal correction to be issued which should be avoided.**
**Please note, changes to authorship (addition or removal) will not be allowed once the manuscript has been formally accepted for publication.**

**For papers containing one or more consortia, all members of the consortium who contributed to the paper must be listed in the paper (i.e., print/online PDF). If necessary, individual authors can be listed in both the main author list and as a member of a consortium listed at the end of the paper. When submitting your revised manuscript via the online submission system, the consortium name should be entered as an author, together with the contact details of a nominated consortium representative. See our authorship policy and further consortia formatting guidelines, which should be adhered to prior to acceptance.**

**2. PUBLICATION REQUIREMENTS:**

**Pending submission of your final files, you will be contacted following the acceptance of your manuscript to complete the relevant publication and payment forms for your manuscript, hence you do not need to return any such forms with your final files.**

**3. ORCID:**

**Cell Death & Disease is committed to improving transparency in authorship. As part of our efforts in this direction, we are now requesting that all authors identified as 'corresponding author' create and link their Open Researcher and Contributor Identifier (ORCID) with their account on the Manuscript Tracking System (MTS) prior to acceptance. ORCID helps the scientific community achieve unambiguous attribution of all scholarly contributions. For more information please visit http://www.springernature.com/orcid**

**For all corresponding authors listed on the manuscript, please follow the instructions in the link below to link your ORCID to your account on our MTS before submitting the final version of the manuscript. If you do not yet have an ORCID you will be able to create one in minutes. https://www.springernature.com/gp/researchers/orcid/orcid-for-nature-research.**

**IMPORTANT: All authors identified as 'corresponding author' on the manuscript must follow these instructions. Non-corresponding authors do not have to link their ORCIDs but are encouraged to do so. Please note that it will not be possible to add/modify ORCIDs at proof. Thus, if they wish to have their ORCID added to the paper they must also follow the above procedure prior to acceptance.**

To support ORCID's aims, we only allow a single ORCID identifier to be attached to one account. If you have any issues attaching an ORCID identifier to your MTS account, please contact the Platform Support Helpdesk.

You can now use a single sign-on for all your accounts, view the status of all your manuscript submissions and reviews, access usage statistics for your published articles and download a record of your refereeing activity for the Nature journals.

Sincerely,

Ygal Haupt
Receiving Editor
Cell Death & Disease

------------------------------------------------------------------------------------

Reviewer Comments:

This email has been sent through the NPG Manuscript Tracking System NY-610A-NPG&MTS

*Confidentiality Statement:*

*This e-mail is confidential and subject to copyright. Any unauthorised use or disclosure of its contents is prohibited. If you have received this email in error please notify our Manuscript Tracking System Helpdesk team at http://platformsupport.nature.com .*

*Details of the confidentiality and pre-publicity policy may be found here http://www.nature.com/authors/policies/confidentiality.html*

Privacy Policy | Update Profile

## 回复：转发：Decision letter for CDDIS-21-2401RR: Accepted in Principle (change of authorship)

1封邮件

<jinxinxy2@csu.edu.cn> 今天 早上8:54

收件人： jinxinxy2@csu.edu.cn <jinxinxy2@csu.edu.cn>

yes，i agree
xin jin

发自PORSCHE DESIGN HUAWEI Mate 40

-------- 原始邮件 --------
发件人： jinxinxy2@csu.edu.cn
日期： 2022年3月11日周五 早上8:54
收件人： jinxinxy2@csu.edu.cn
主 题： 转发：Decision letter for CDDIS-21-2401RR: Accepted in Principle (change of authorship)

Dr. XIN JIN

The manuscript entitiled "The RBPJ/DAPK3/UBE3A signaling axis induces PBRM1 degradation to modulate the sensitivity of renal cell carcinoma to CDK4/6 inhibitors" has been submited to *Cell Death & Disease*. The original antuor list is "Wentao Liu, Bin Zhang, Dan Zhang, Feng Guo, Kun Ye, Liang Zhu, and Xin Jin". Dr.Dan Zhang performed a lot of experimental works in the revision stage of the article. Bases on her contribution to the article, we decided to add her to the article. The new author list is :Wentao Liu, Bin Zhang, Dan Zhang, Feng Guo, Kun Ye, Liang Zhu, and Xin Jin. If you agree/disaggree the change, please response me you decision.

Thanks
Xin Jin

-------- 原始邮件 --------
发件人： cddisease@springernature.com
日期： 2022年3月3日周四 傍晚5:11
收件人： jinxinxy2@csu.edu.cn
主 题： Decision letter for CDDIS-21-2401RR: Accepted in Principle
Manuscript Number: CDDIS-21-2401RR
Title: The RBPJ/DAPK3/UBE3A signaling axis induces PBRM1 degradation to modulate the sensitivity of renal cell carcinoma to CDK4/6 inhibitors
Authors: Wentao Liu, Bin Zhang, Dan Zhang, Feng Guo, Kun Ye, Liang Zhu, and Xin Jin

Dear Dr Jin,

I am very pleased to inform you that your manuscript CDDIS-21-2401RR entitled "The RBPJ/DAPK3/UBE3A signaling axis induces PBRM1 degradation to modulate the sensitivity of renal cell carcinoma to CDK4/6 inhibitors" has been **provisionally accepted** for publication in *Cell Death & Disease*.

**PLEASE READ THE FOLLOWING CAREFULLY**

Please note that we won't be able to officially confirm acceptance of your manuscript and proceed with the publication process until the final version of your manuscript has been submitted as per the instruction below.

*Cell Death & Disease* is a fully open access journal. Articles are made freely accessible on publication under a http://creativecommons.org/licenses/by/4.0"" target=""_blank""> CC BY license (Creative Commons Attribution 4.0 International License). This license allows maximum dissemination and re-use of open access materials and is preferred by many research funding bodies. For further information about article processing charges, open access funding, and advice and support from Nature Research, please visit https://www.nature.com/cddis/open-access

At acceptance, you will be provided with instructions for completing this CC BY license on behalf of all authors. This grants us the necessary permissions to publish your paper. Additionally, you will be asked to declare that all required third party permissions have been obtained, and to provide billing information in order to pay the article-processing charge (APC).

For more detailed information please check the https://www.nature.com/documents/cddis-gta.pdf"> Guide to the Authors.

**1. SUBMITTING YOUR FINAL FILES:**

Please submit your final manuscript here:
https://mts-cddis.nature.com/cgi-bin/main.plex?el=A6CA1Unx2C7BtFU7I3A9ftdain5ppQ62KFzO9JNgRP0wZ

- Files must not contain highlighting or tracked changes.
- Cell Death & Disease references should follow the Vancouver format. All authors should be listed for papers with up to six authors; for papers with more than six authors, the first six only should be listed, followed by et al.
- Figure and table legends must be included at the end of article file, after the references.
- Figure files must be uploaded as individual files and then included in the merged file, ensuring that figures with multiple panels are uploaded as one file (e.g. Fig. 1A, 1B uploaded as Fig. 1).
- Tables must be editable and either uploaded as separate files or included in the article file, after the references.
- All supplementary information, including supplementary figure and table legends, must be uploaded separately and not included in the article file.
- An Author Contribution Statement must be provided.
- All authors identified as 'corresponding author' should create and link their Open Researcher and Contributor Identifier (ORCID) with their account on the Manuscript Tracking System (MTS) prior to

acceptance.

Please note that you must retain a copy of all uncropped **original experimental data at high resolution for five years, to be shown upon request by the journal. Failure to produce such data on request can result in a retraction by the journal.**

**- AUTHOR LIST:**
**Please review the author list to verify that it is complete and accurate. We ask that you consult with your co-authors to ensure that all names, affiliations, and titles are represented correctly. Should such details need correcting after publication, this will require a formal correction to be issued which should be avoided.**
**Please note, changes to authorship (addition or removal) will not be allowed once the manuscript has been formally accepted for publication.**

**For papers containing one or more consortia, all members of the consortium who contributed to the paper must be listed in the paper (i.e., print/online PDF). If necessary, individual authors can be listed in both the main author list and as a member of a consortium listed at the end of the paper. When submitting your revised manuscript via the online submission system, the consortium name should be entered as an author, together with the contact details of a nominated consortium representative. See our authorship policy and further consortia formatting guidelines, which should be adhered to prior to acceptance.**

**2. PUBLICATION REQUIREMENTS:**

**Pending submission of your final files, you will be contacted following the acceptance of your manuscript to complete the relevant publication and payment forms for your manuscript, hence you do not need to return any such forms with your final files.**

**3. ORCID:**

**Cell Death & Disease is committed to improving transparency in authorship. As part of our efforts in this direction, we are now requesting that all authors identified as 'corresponding author' create and link their Open Researcher and Contributor Identifier (ORCID) with their account on the Manuscript Tracking System (MTS) prior to acceptance. ORCID helps the scientific community achieve unambiguous attribution of all scholarly contributions. For more information please visit http://www.springernature.com/orcid**

**For all corresponding authors listed on the manuscript, please follow the instructions in the link below to link your ORCID to your account on our MTS before submitting the final version of the manuscript. If you do not yet have an ORCID you will be able to create one in minutes. https://www.springernature.com/gp/researchers/orcid/orcid-for-nature-research.**

**IMPORTANT: All authors identified as 'corresponding author' on the manuscript must follow these instructions. Non-corresponding authors do not have to link their ORCIDs but are encouraged to do so. Please note that it will not be possible to add/modify ORCIDs at proof. Thus, if they wish to have their ORCID added to the paper they must also follow the above procedure prior to acceptance.**

To support ORCID's aims, we only allow a single ORCID identifier to be attached to one account. If you have any issues attaching an ORCID identifier to your MTS account, please contact the Platform Support Helpdesk.

You can now use a single sign-on for all your accounts, view the status of all your manuscript submissions and reviews, access usage statistics for your published articles and download a record of your refereeing activity for the Nature journals.

Sincerely,

Ygal Haupt
Receiving Editor
Cell Death & Disease

--------------------------------------------------------------------------------

Reviewer Comments:

This email has been sent through the NPG Manuscript Tracking System NY-610A-NPG&MTS

*Confidentiality Statement:*

*This e-mail is confidential and subject to copyright. Any unauthorised use or disclosure of its contents is prohibited. If you have received this email in error please notify our Manuscript Tracking System Helpdesk team at http://platformsupport.nature.com .*

*Details of the confidentiality and pre-publicity policy may be found here http://www.nature.com/authors/policies/confidentiality.html*

Privacy Policy | Update Profile
